# Anti-Hebbian plasticity drives sequence learning in striatum

**DOI:** 10.1038/s42003-024-06203-8

**Published:** 2024-05-09

**Authors:** Gaëtan Vignoud, Laurent Venance, Jonathan D. Touboul

**Affiliations:** 1 Center for Interdisciplinary Research in Biology (CIRB), College de France, CNRS, INSERM, Université PSL, Paris, France; 2 Department of Mathematics and Volen National Center for Complex Systems, Brandeis University, Waltham, MA USA

**Keywords:** Learning and memory, Computational neuroscience

## Abstract

Spatio-temporal activity patterns have been observed in a variety of brain areas in spontaneous activity, prior to or during action, or in response to stimuli. Biological mechanisms endowing neurons with the ability to distinguish between different sequences remain largely unknown. Learning sequences of spikes raises multiple challenges, such as maintaining in memory spike history and discriminating partially overlapping sequences. Here, we show that anti-Hebbian spike-timing dependent plasticity (STDP), as observed at cortico-striatal synapses, can naturally lead to learning spike sequences. We design a spiking model of the striatal output neuron receiving spike patterns defined as sequential input from a fixed set of cortical neurons. We use a simple synaptic plasticity rule that combines anti-Hebbian STDP and non-associative potentiation for a subset of the presented patterns called rewarded patterns. We study the ability of striatal output neurons to discriminate rewarded from non-rewarded patterns by firing only after the presentation of a rewarded pattern. In particular, we show that two biological properties of striatal networks, spiking latency and collateral inhibition, contribute to an increase in accuracy, by allowing a better discrimination of partially overlapping sequences. These results suggest that anti-Hebbian STDP may serve as a biological substrate for learning sequences of spikes.

## Abstract (web summary)

Mathematical models and computer simulations suggest that anti-Hebbian STDP, the type of plasticity observed between cortex and striatum, could support the learning of sequences in the brain.

## Introduction

Nerve cells generate spatio-temporal patterns of action potentials, generally construed to convey information in the central nervous system. While spiking sequences have indeed been observed on a variety of timescales and in distinct brain areas^1–7^, the biological mechanisms employed to encode, store a sequence or distinguish between different sequences are still largely unknown. At behavioral timescales (seconds), episodic experience is by nature a sequence of events^8^. In the brain, this results in the generation of spatio-temporal spike sequences, as for instance with hippocampal place cells activating following the movement of the animals^1^ or with action-dependent spike sequences emerging in a virtual navigation-decision task in parietal cortex^2^. Generating dynamical output also requires the formation of sequential cortical activity, as observed in bird’s ability to repeat spatio-temporal sequences over tens of seconds and with temporal structure maintaining millisecond accuracy within synfire chains^3^, or more generally the generation of sequential activation of neural assemblies^4^. At shorter timescales, cortical spike sequences lasting tens of milliseconds were also reported in the relative timing of spikes between sequences in oscillating neural assemblies, where spike ordering and latencies depended on the stimulus^5^, in sequential activation after an up state transition^6^ in response to a single spike^9^, or even in spontaneous patterns of activity^10^. Theoretically, networks with Hebbian synaptic plasticity have the ability to generate sequential activity or to complete sequences they have been exposed to^11–15^.

Interpreting this ubiquitous sequential spike activity requires neural mechanisms to identify and distinguish sequences, so output neurons may fire in response to specific patterns and remain silent for others. Identifying a sequence is a complex task that requires integrating signals on a timescale of several spikes. Moreover, it requires distinguishing sequences that share similar sub-patterns, for instance sequences that are initially identical and only differ in their last spikes; the learning of such overlapping sequences could even sometimes appear incompatible.

Machine learning algorithms were proposed for the selection of spike sequences^16^. In that domain, a large body of work has addressed the problem of generating a specific target output spike train in response to a sequence of spikes. Methods proposed relied on error backpropagation^17^, high-threshold projection^18^, Remote Supervision Methods (ReSuMe)^19^, or, for the Chronotron^20^, smooth modifications of the Victor & Purpura distance for spike trains to compute error terms, were shown to be successful in performing those tasks. Closer to the problem at hand, some algorithms were designed to decode statistical information from spike trains, or even to simply spike in response to particular sequences of input spikes. Those techniques, which include the Tempotron^21,22^, and its extensions^16,23^, were designed to discriminate specific structures of spike sequences (in particular, patterns defined by the latency between pre-synaptic neurons spikes or synchrony) and rely on a computational learning rule that potentiates synapses associated with specific (rewarded) patterns when the neurons did not spike, and depress synapses of non-rewarded patterns if the output neuron spiked in response to the pattern. The accuracy of the tempotron was then estimated by the fraction of rewarded pattern presentations associated with a spike fired at any point during a sequence. These early work provide a solid basis for testing sequence learning that we use and extend here.

Our study takes an opposite approach to machine learning and computational algorithms that looked for efficient algorithms to process sequences with spiking neurons. Here, we model the biological learning rules observed in the striatum and explore the type of patterns that can be learned from them. The dorsal striatum, the main input structure of the basal ganglia, receives excitatory inputs from all cortical areas and most thalamic nuclei^24^ and has been shown to play a major role in action selection^25–28^ and to be a prominent site for memory formation and procedural learning^28^. In this variety of tasks, it is expected that the striatum uses information from sequences of evidence to take a decision^29–31^. Contrasting with associative recurrent cortices that are efficient in recollecting missing information when presented with partial patterns, the striatum is a largely feedforward network, that combines a variety of cortical inputs to produce an output. Corticostriatal synapses display anti-Hebbian Spike Timing Dependent Plasticity (STDP) in vitro^32–36^ or in vivo^37,38^, whereby a cortical spike followed by a striatal medium spiny neuron (MSN) spike leads to a depression of the associated synaptic weight. While many computational studies have investigated the impact of Hebbian STDP, only a few studies considered anti-Hebbian STDP. Those concentrated on the question of stability of synaptic weights^39–41^, compensation of dendritic attenuation^42^, cancellation of correlated signals and novelty detection^43,44^, in particular in the electrosensory system of the mormyrid electric fish. As shown in all these works, when presented with correlated activity, anti-Hebbian STDP leads to the depression of the associated synapses. This phenomenon could naturally endow the system with the patience necessary to listen to full sequences and identify specific ones.

Our study explores the possible role of cortico-striatal anti-Hebbian STDP, reported experimentally in regions typically associated with procedural learning^36^, in the learning of spike sequences. We use a theoretical and computational approach. To test for different features separately, our models progress from the simplest to more realistic, which allows an in-depth exploration of the ability of the biological learning rules to support sequence learning and the role played by each biological feature in contributing to sequence learning. Our results show that three basic biological mechanisms observed in the striatum, namely anti-Hebbian learning, spike latency and collateral inhibition, combined with a reward mechanism, are particularly efficient for the learning of spatio-temporal spiking sequences. In particular, we show that the combination of anti-Hebbian STDP with a simple, non-associative LTP is sufficient for a single MSN to acquire the ability to distinguish sequences, providing a functional relevance for the anti-Hebbian STDP learning rules recently observed. Moreover, while the simplest models of neurons with instantaneous firing can learn sequences, our simulations show that they tend to spike too early, which is problematic in particular when learning to distinguish overlapping spike sequences. We show that this drawback is naturally corrected by incorporating spike latency and collateral inhibition, two key classical biological observations from the striatal network. This analysis further proposes a functional role for these two biological observations in the framework of sequence learning, suggesting that they could contribute to a remarkable ability to identify and optimize the learning of sequences of spikes that outperforms some artificial learning algorithms subjected to similar constraints.

## Results

### Modeling a sequence learning task in striatum

Given a spatio-temporal sequence of cortical spikes, as observed in vivo in rodents or non-human primates^31,45–50^, we posit that a MSN has learned to distinguish between two groups of sequences if it acquires the ability to selectively spike at or after the end of a subset of sequences and remain silent otherwise. While basic, this notion of sequence learning is quite distinct from the literature. Indeed, previous works focused on the ability of neurons to (i) reproduce or complete a target spike train^17–20^ or (ii) classify a pattern by spiking at any time during the presentation of the stimulus^16,21–23^. Our notion of sequence learning is similar to the latter criterion. However, imposing the MSN to spike after the end of the sequence allows the learning of complete sequences and endows the system with distinguishing nested stimuli. In detail, for any pattern A eliciting a spike, any superpattern of A (i.e., spike pattern containing A) needs to be in the same class for the learning task to be consistent. Requiring instead that the MSN spikes at the end of a pattern presentation opens the way to distinguish such patterns and respond differently to each of them, thereby allowing to exploit any additional information contained in a superpattern. From a functional viewpoint, task (ii) as well as our task (namely, requiring the MSN to fire after specific patterns) both relate to the role of the striatal neurons that integrate cortical correlated patterns and then spike to trigger further downstream pathways leading to motor processing and eventually, an action.

We explored the ability of striatal networks to do this task using simple, yet increasingly realistic, mathematical models. MSNs integrate numerous cortical and thalamic inputs, and act as coincidence detectors, since their high threshold requires the concomitant arrival of many spikes to induce a spike, which is fired after a period of latency^51,52^. Those neurons have been described in depth, both biologically and computationally, and several mathematical models were proposed to describe their behavior^53–57^. Within the striatum, MSNs produce sparse inhibitory collateral connections among themselves, which reportedly plays a major role in the regulation of MSN firings or their overall activity ^58–63^.

Our approach consists of starting from the most simple setting of a single MSN receiving many cortical inputs and expressing the type of cortico-striatal plasticity observed experimentally, and building our way towards more complex models of two-neuron networks with non-linearities and adaptation, assessing in each case the performance of the system. All models are defined in the “Material and Methods” section.

**Fig. 1:**
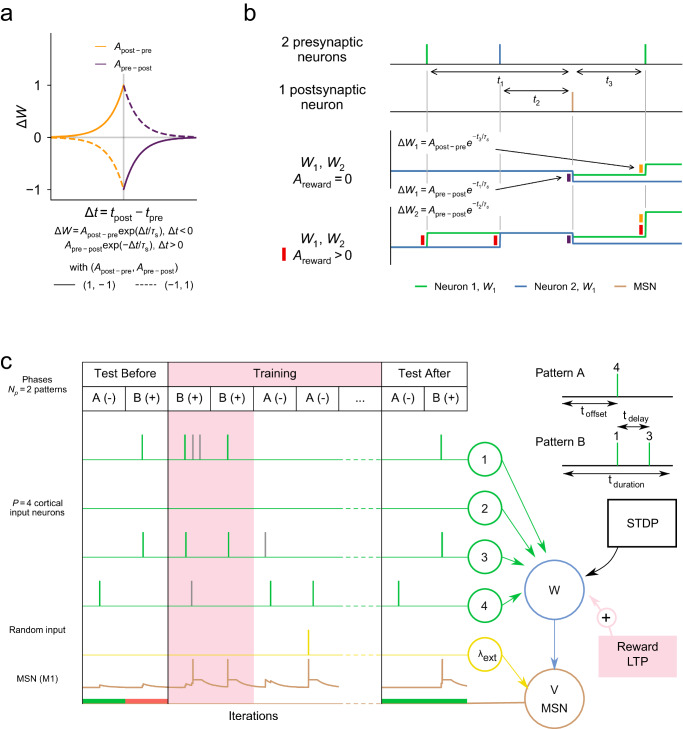
Computational framework. **a** Four types of STDP. Synaptic weight updates Δ*W* as a function of Δ*t* are piecewise branches of exponential with time constant *τ*_*s*_ and amplitude ${A}_{{{{{{{\rm{post-pre}}}}}}}}$ for post-pre pairings (Δ*t* < 0, orange) and *A*_pre-post_ for pre-post pairings (Δ*t* > 0, purple); asymmetric Hebbian STDP (dashed line) and anti-Hebbian STDP (solid line). **b** Non-associative potentiation to represent reward signals. Example of synaptic updates resulting from spike train from two input neurons (top, neuron 1 in green, neuron 2 in blue) and one output neuron (second line, in brown). Synaptic update resulting from asymmetric anti-Hebbian STDP (third line) or with non-associative reward-LTP (red, bottom line). **c** Classification task. Schematic representation of the striatal network (right) with *P* = 4 cortical neurons (green), a random input neuron with rate *λ*_ext_ (yellow) and one MSN, represented by its membrane potential *V* (brown). Two mechanisms of synaptic plasticity are considered in the dynamics of the synaptic weight *W* (blue): STDP and LTP related to the reward signal (reward-LTP) (red). Example of the learning task (left), with test sessions and the training protocol (middle). *N*_*p*_ = 2 patterns *A* and *B* are presented to the network, with *A* being non-rewarded (−) and *B* rewarded (+). Each pattern represent sequential activity (top right), *A* with a single cortical spike and *B* with two spikes separated by a delay *t*_delay_. All patterns have a duration of *t*_duration_, and correlated cortical spikes are presented at *t*_offset_. Spiking activity of the cortical neurons (green for pattern spikes and gray for random spikes) and the random input neuron (yellow) are represented along with the membrane potential *V* of the output neuron (MSN, M1, brown). In the test sessions, below the MSN potential, are represented accuracy results (correct classification in green, wrong classification in red).

We started by modeling the neuron as a linear integrate-and-fire neuron (M1), with parameters fitted from electrophysiological recordings of MSNs (*n* = 16) in acute brain slices of adult mice (Material and Methods and Fig. S1). Figure S1a provides a comparison between this experimental data (summarized in Table 1) and the model, and Fig. S1b compares the neuron’s parameters with other models reported in the literature^53,56^. We obtained a model that reproduced relatively accurately the phenomenology of MSN activity; model M1 thus fitted however failed to reproduce the firing rates observed experimentally in response to a constant input, which should not affect the results of our learning experiments since these are only related to single spike responses to transient spiking input. The cortico-striatal synapses were endowed with both STDP^32,33^ (Fig. 1a) and reward-LTP (Fig. 1b). To emulate learning, patterns of spiking activity were presented to the MSN during the training phase. A rewarded pattern was deemed learned if the MSN fired after the presentation of the whole pattern, whereas non-rewarded patterns should not elicit any spike. Before learning, patterns *A* and *B* did not trigger any spikes of the MSN, leading to a correct classification for *A* (as a non-rewarded pattern) and a misclassification for *B*. After learning, pattern *A* still did not elicit any spike while the MSN emitted a spike after the presentation of all cortical spikes of pattern *B*, leading to a correct classification (Fig. 1c). The accuracy of the learning process was estimated through the averaged numbers of correct responses to the different patterns.

**Table 1:** Numerical parameter values for MSN models M1 and M2

Parameters	fitted M1	M2 from ref. ^53^	M1 from ref. ^56^
*V*_eq_ (mV)	−76.72	−80	−80
*V*_th_ (mV)	−39.51	−20	−45
*V*_*r*_ (mV)	−41.70	−55	−80
*R* (MΩ)	118.50	100	80
*τ* (ms)	11.85	n/a	16
*C* (nF)	0.098	0.05	0.2
*a* (ms^−1^)	n/a	0.01	n/a
*b* (nF ms^−1^)	n/a	−0.02	n/a
*d* (nF ms^−1^ mV)	n/a	0.15	n/a

Parameters for Models M1 and M2 fitted to the electrophysiological data and compared to the parameters of Model (M1) used in ref. ^56^.

### Weights distribution and firing rates in response to Poisson inputs

In a first step towards understanding the role of STDP and reward-LTP on learning, we analyzed the behavior of the system when presented with various inputs and endowed with different plasticities (Fig. 1a). To quantify the performance of sequence learning, we compared the evolution of the following types of STDP:

- Symmetric anti-Hebbian STDP (symmetric LTD): ${A}_{{{{{{{\rm{post-pre}}}}}}}}={A}_{{{{{{{\rm{pre-post}}}}}}}}=-1$, which represents STDP where correlated spiking only leads to depression of the synaptic weight.
- Asymmetric Hebbian STDP: ${A}_{{{{{{{\rm{post-pre}}}}}}}}=-1$ and *A*_pre-post_ = 1, where pre-post pairings lead to potentiation, while post-pre pairings lead to depression.
- Asymmetric anti-Hebbian STDP: ${A}_{{{{{{{\rm{post-pre}}}}}}}}=1$ and *A*_pre-post_ = − 1, which is the reverse of the asymmetric Hebbian STDP.
- Symmetric Hebbian STDP (Symmetric LTP): ${A}_{{{{{{{\rm{post-pre}}}}}}}}={A}_{{{{{{{\rm{pre-post}}}}}}}}=1$, which represents STDP where correlated spiking only leads to potentiation of the synaptic weight.

**Fig. 2:**
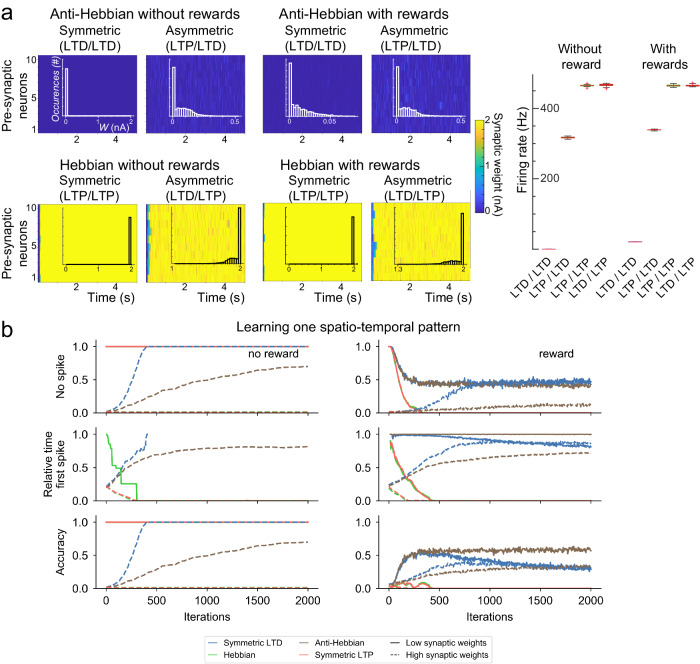
Learning of Poisson sequences with STDP and non-associative reward-LTP. **a** Statistics of synaptic weights and firing rates in response to stationary Poisson input. Dynamics of synaptic weights (heatmap), distribution of weights (overlaid histogram), and output firing rate statistics (right boxplot) for Hebbian and anti-Hebbian symmetric or asymmetric STDP, resulting from Poisson input with *P* = 10 cortical neurons firing as Poisson processes with a rate of 100Hz (high rates chosen to display the impact of a high number of spike pairings, similar results were obtained for 10 Hz input firing rates, see Fig. S2). All cases show convergence of the weights to a stationary distribution. Anti-Hebbian rules led to low weights and moderate output firing rates sensitive to the presence of reward-LTP (two-sample *t* test between any anti-Hebbian firing rate and any of the other conditions were associated with high significances, *p* < 0.0005), while Hebbian rules showed saturation of the synaptic weights with high firing rates and no sensitivity to the type of plasticity or the possible presence of reward-LTP (*p* > 0.1). Boxplots of the stationary distribution combine all values of the synaptic weights after 5s for all neurons. **b** Dynamics of learning with a Poisson sequence. Different learning properties, during the presentation of a Poisson sequence, with *P* = 10 cortical neurons, without (left) or with (right) non-associative reward-LTP, for different types of STDP rules (color) and different values of initial synaptic weights (low in solid lines, high in dashed lines). (top) Probability to observe no spike during pattern presentation, (middle) relative timing of the first post-synaptic spike, (bottom) accuracy. (Note: the traces from symmetric LTP and asymmetric Hebbian STDP are superimposed.) Synaptic weights initialized uniformly at random in the [0., 0.05] nA range; *A*_reward_ = 0.5; *N* = 500 independent networks; Membrane potential and plasticity reset between each pattern.

We distinguished Hebbian learning rules (symmetric and asymmetric Hebbian STDP), characterized by *A*_pre-post_ = 1, from anti-Hebbian learning rules (symmetric and asymmetric anti-Hebbian STDP) with *A*_pre-post_ = − 1 (Fig. 1a). In each situation, we compared learning accuracies with or without reward LTP (Fig. 2).

We presented the MSN with random activity from the cortical neurons (firing as Poisson processes with rates of 10 Hz or 100 Hz for each cortical neuron) and studied the distribution of the synaptic weights (Fig. S2 or 2a). A first observation arising from these simulations is the fact that Hebbian rules typically lead to an overall potentiation of the synaptic weights, with symmetric LTP systematically leading to a saturation of the synaptic weights, a consequence of the well-known divergence of synaptic weights in Hebbian theory^64–66^. This resulted in a saturating firing rate for the MSN that showed no dependence in whether or not patterns were rewarded (symmetric LTP without rewards in response to 10  or 100 Hz Poisson input yielded MSN median firing rates of 89.26  and 465.15 Hz, respectively, Hebbian without rewards 90.07  and 467.32 Hz, symmetric LTP with rewards 88.38 and 467.90 Hz, and Hebbian with rewards 89.27 or 466.37 Hz, two-sample t-test with 20 repetitions found no significant difference between any of these conditions, *p* > 0.1). Instead, both of the anti-Hebbian rules rather led to stationary distributions of synaptic weights remaining away from saturation, and with a steady fraction of weights having very low values. Without reward-LTP, symmetric LTD led to a decrease in the synaptic weight until the MSN became silent. Reward-LTP prevented such an extinction of the activity and yielded a distribution of synaptic weights concentrated on relatively low values. Asymmetric anti-Hebbian STDP yielded similar results in both cases. For very low initial values of the synaptic weights, rare extinctions of the network are possible, but in most cases, we observed the convergence of the synaptic weights to a non-trivial distribution of weights, independent of the initialization, with a typical profile showing a (dynamically-varying) fraction of the weights being extinct while others were distributed on support away from the saturation threshold. The distribution profiles with or without reward-LTP were found to be similar, with, expectedly, broader support and larger synaptic weights reached in the presence of reward LTP. Altogether, this analysis shows that, in contrast with Hebbian rules well known to be prone to divergences in the synaptic weights, anti-Hebbian rules typically allow maintaining low synaptic weights, possibly leading to the extinction of the network (absence of MSN spike) in the absence of reward-LTP. With these rewards, both anti-Hebbian rules reached a steady distribution of weights with relatively low amplitudes and the MSN’s activity stabilizes at a steady, relatively sparse activity that increases frequency upon application of rewards (for 10 and 100 Hz, respectively, symmetric LTD went from 0.00 and 0.04 Hz without rewards to 9.91 and 11 Hz with rewards, or for asymmetric anti-Hebbian learning, firing rates going from 0.01  and 0.52 Hz without rewards to 22.37  and 97 Hz with rewards. Two sample *t* test between any anti-Hebbian STDP and any other condition showed a significant difference with a *p* < 0.0005.).

### Learning rules and mechanisms of learning a single sequence

We then investigated the dynamics of the system in response to a single pattern, obtained as a Poisson process with intensity *λ*_poisson_ = 1 kHz on a time interval of duration *t*_poisson_ = 5 ms, conditioned with having at least two spikes (Fig. 2b). We computed the probability that the MSN remains silent, the relative timing of the first spike of the MSN and the resulting accuracy, for both non-rewarded and rewarded patterns, and closely investigated the impact of the plasticity rules and rewards on the synaptic weights.

To understand the mechanisms by which anti-Hebbian learning yielded accurate behaviors, we considered the heuristically the impact of the repeated presentation of a pattern on synaptic weights and firing or quiescence of an MSN, for non-rewarded or rewarded patterns, and for synapses expressing one type of Hebbian or anti-Hebbian plasticity. For non-rewarded patterns, two different situations arise depending on initial conditions on synaptic weights:

- if weights are low enough so that the pattern presentation does not induce a spike, then the absence of any plasticity (no reward-LTP and no synaptic weight update because of the quiescence of the MSN) implies that weights remain unchanged and a future pattern presentation will not lead to any spike if weights were not modified by another process in the meantime (Fig. 2b).
- If synaptic weights are initially large enough to trigger a spike in the MSN in response to this non-rewarded pattern, then the pre-post depression of anti-Hebbian STDP (both present in symmetric and asymmetric plasticities) will lead to a decay of the synaptic weights associated with the pattern ultimately leading the MSN to stop firing. Instead, Hebbian STDP will only reinforce the initial MSN spiking by making the synaptic weights of the pre-synaptic neurons that spiked before the MSN larger, preventing the neuron to stop firing in response to that non-rewarded pattern (Fig. 2b).

We next considered a rewarded pattern that, initially, does not induce an MSN spike. In that case, all synapses associated with a spike during the pattern presentation are potentiated through reward-LTP. The repeated presentation of the pattern thus ultimately leads to an MSN spike, regardless of plasticity. Once the MSN spikes, STDP combines with the rewards and produce distinct outcomes depending on the type of plasticity. For Hebbian STDP (both symmetric LTP and asymmetric Hebbian STDP), the pre-post potentiation further increases the synaptic weight of pre-synaptic neurons leading the neuron to spike increasingly early as the pattern is presented (Fig. 2b), and providing an incorrect learning of the sequence. Contrasting with this effect, anti-Hebbian STDP endows the system with mechanisms allowing the MSN to spike at the end of the pattern (see statistics in Fig. 2b). Typically, when *A*_reward_ is small, the repeated presentation of a rewarded pattern in the absence of an MSN spike occurs at the end of the sequence (a success in our task). But even if the spike does not arise at the end of the sequence, pre-post depression and post-pre potentiation have generally the effect of moving the spike to the end of the pattern. Indeed, because *A*_reward_ + *A*_pre-post_ < 0, the synaptic weights of pre-synaptic neurons spiking just before the MSN decreases, eventually preventing the neuron from spiking and moving the spike to later in the pattern where post-pre potentiation is favorable to support a spike. While these mechanisms seem to favor the learning of sequences and MSN firing at the end of the pattern, they also allow two modes of failure depending on pattern duration and synaptic weights thresholds: a decay of synaptic weights before the MSN spike, and a possible increase in the synaptic weights of spikes arising early in the pattern.

**Fig. 3:**
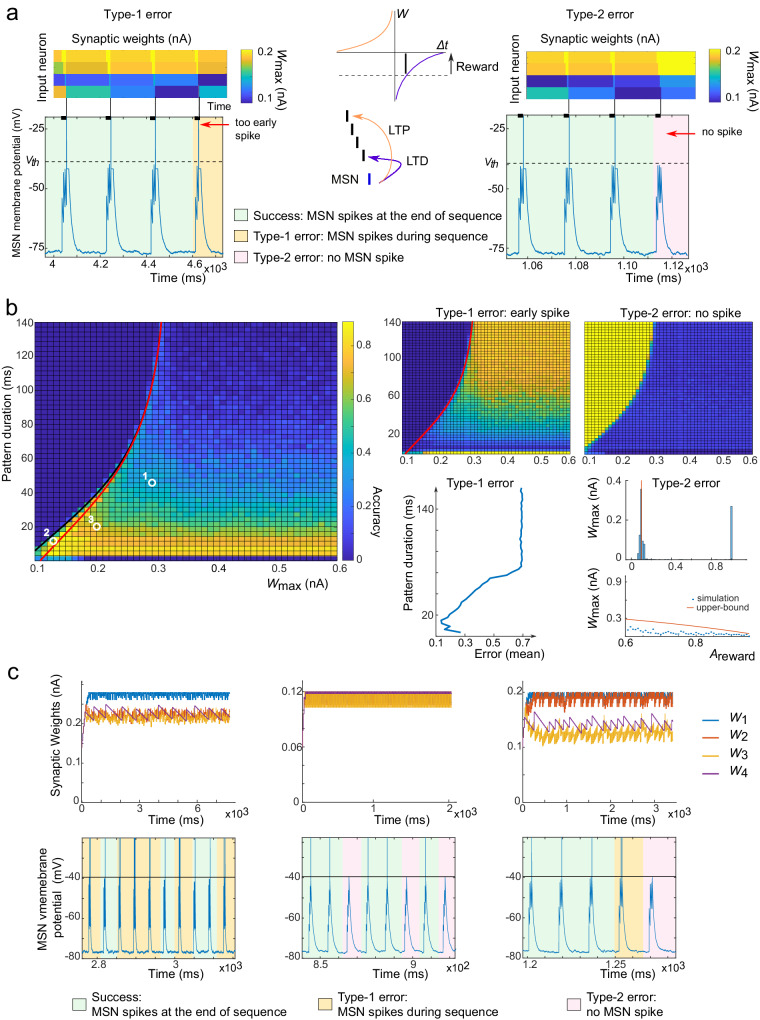
Impact of anti-Hebbian learning rules and non-associative reward-LTP on synaptic weights in learning sequences. *P* = 10 pre-synaptic neurons, 4 spikes per pattern with a constant inter-spike interval *T*_delay_ of varying duration. MSN model M1, and anti-Hebbian STDP at all synapses. **a** Center: successful response to a rewarded pattern leads to potentiation of all the synapses of neurons firing earlier than ${T}_{p}={\tau }_{s}\log (-\frac{{A}_{{{{{{{\rm{pre-post}}}}}}}}}{{A}_{{{{{{{\rm{reward}}}}}}}}})$ before the end of the pattern (black line), while others get depressed. Repeated successes may thus lead to one of two failures: type 1 errors (left, orange) where potentiation of early cortical spikes accumulate and lead the MSN to spike before the end of the sequence (as visible after 3 successes on the left, with the visible increase in *W*_3_ after the third pattern leads a spike after the third pattern at the subsequent presentation of the pattern); or type 2 error (right, pink) where depression of the synaptic weights of the last neurons spiking in the pattern leads to an absence of spiking. (in both cases, default parameters with a pattern duration of 20 ms). **b** Accuracy as a function of pattern duration (ordinate) and synaptic weight threshold ${w}_{\max }$ (fraction of successes out of 200 pattern presentations), as well as the complementary fractions of type 1 and type 2 errors (right). As expected, long patterns with low synaptic thresholds lead favor type 2 errors (see also mean type 1 error frequency as a function of pattern duration), while high thresholds and long patterns are associated with more frequent type 1 errors. Red curve: necessary condition for the emergence of type 1 error and Black curve: necessary condition for the emergence of a spike (above this curve, no spike is fired and all trajectories are associated with an absence of spike, or type 2 error). The maximal frequency of type 2 error, as computed in the main text, provides a fair estimate of the order of magnitude of the occurrence of type 1 errors over the whole heatmap (right histogram below the heatmap) and a clear upper bound for the occurrence of type 1 errors when varying *A*_reward_ (below). **c** Three typical learning situations for high threshold and long patterns (position 1 in **b**), low thresholds and durations (position 2 in **b**), and intermediate threshold and durations (position 3 in **b**). We observe the distinct occurrences of error types in these distinct situations, as well as a typical serrated pattern for the last synaptic weight in the sequence corresponding to sequences of successes (decays) interspersed with a failure leading to a sudden increase.

To examine further the mechanisms by which this learning occurs, we further simplified the setup by considering a simple pattern composed of four spikes with equal inter-spike intervals in the absence of Poisson external spikes unrelated to the pattern (Fig. 3). When success in the task arose and the MSN spiked at the end of the pattern, all synaptic weights associated with neurons spiking towards the end of the pattern (precisely, less than ${T}_{p}={\tau }_{s}\log (-\frac{{A}_{{{{{{{\rm{pre-post}}}}}}}}}{{A}_{{{{{{{\rm{reward}}}}}}}}})$ units of time before the end of the pattern) are depressed, with the strongest depression for the neuron firing last in the pattern. Instead, neurons firing more than *T*_*p*_ units of the time before the end of the pattern see their synaptic weight being potentiated (Fig. 3a) since the amount of reward received exceeds the pre-post LTD. Both phenomena conspire to prevent the emergence of an uninterrupted sequence of successes. Instead, a sequence of successes terminates either :

1. When the accumulated potentiation of synapses early in the pattern exceeds a critical value and leads the MSN to spike before the end of the sequence (type 1 error in Fig. 3a, left panel), or
2. When the decay of synaptic weights associated late in the sequence eventually causes the MSN to fail to spike in response to the pattern (type 2 error in Fig. 3a, right panel).

The potential occurrence of these failure modes depends both on the neuron’s parameters, input timing and duration and any possible bounds on synaptic weights. In the case of the integrate and fire neuron, simple conditions can be found to outline the emergence of one or the other type of error. Consider a pattern with *n* spikes occurring at times (*t*_1_, ⋯  , *t*_*n*_) and denote ${\delta }_{k}^{l}={t}_{l}-{t}_{k}$ the time delay between the kth spike in the pattern and the lth spike. A necessary condition for type 1 error to occur is that the maximal voltage reached before the last spike exceeds the threshold, or ${{V}_{r}}+{{w}_{max}}{{\sum }_{k = 1}^{n-1}}{e}^{-{\delta }_{k}^{n-1}/ \tau } \ge {{V}_{{{{\rm{th}}}}}}$, while a sufficient condition for type 2 errors to systematically occur is that the maximal voltage reached after a full presentation does not exceed the threshold, ${V}_{r}+{w}_{max}{\sum }_{k = 1}^{n}{e}^{-{\delta }_{k}^{n}/\tau }\le {V}_{{{{{{{\rm{th}}}}}}}}$. In the region where both conditions are satisfied, the system presents an alternation of successes, type 1 and type 2 errors that are represented in Fig. 3b for various combinations of synaptic weight thresholds and pattern duration (controlling the delay between consecutive spikes), together with the two conditions derived above (respectively, red and black lines in Fig. 3b). As expected, long durations between spikes favor type 1 errors, while type 2 error appears to be rather uniform on each side of the black curve: arising all the time when thresholds are too low, and otherwise arising occasionally. The occurrence of type 2 error in this region is more dynamic and involves the STDP parameters. Considering that threshold conditions are not saturated, each type 2 error yields a net increase in all synaptic weights of amplitude *A*_reward_, while each success yields, in particular, a decrease in the synaptic weight of the last neuron of the pattern of amplitude ∣*A*_pre-post_ + *A*_reward_∣. This phenomenon yields a typical serrated profile clearly visible in Fig. 3c in the two cases that do not saturate the threshold condition. Between two type 2 errors and if no type 1 error arises, the system cannot support more than $N=\lceil \frac{{A}_{{{{{{{\rm{reward}}}}}}}}}{| {A}_{{{{{{{\rm{pre-post}}}}}}}}+{A}_{{{{{{{\rm{reward}}}}}}}}| }\rceil$ successes. Any error of type 1 arising between two errors of type 2 will only increase the number of pattern presentations between the next type 2 error as a consequence of the post-pre LTP and rewards on the synaptic weight of the last neuron. This implies that *N* provides an upper bound for the frequency of type 2 error and implies that the accuracy cannot exceed 1 − 1/*N*. With our default parameters, *n* = 9, or a type 2 failure rate of around 10%, providing actually a relatively accurate estimate of the stationary rate of the failure across all cases tested in Fig. 3b.

In a generic situation, the anti-Hebbian STDP therefore does not allow reaching a perfect performance, but alternates successes interspersed with failures whose type and rate of occurrence depends on parameters. Various typical situations are shown in Fig. 3c showing frequent type 1 errors for long sequences of rare spikes (left), frequent type 2 errors when synaptic weights are clipped to low maximal values (middle), or alternation of both errors for intermediate parameters (right), and pattern duration alters the ability of the neuron to learn. It is also to smooth out these dynamic alternations of successes and errors that we introduced the MaxAccuracy measure.

In conclusion, only symmetric and asymmetric anti-Hebbian STDP correctly learned to classify rewarded and non-rewarded patterns, whereas Hebbian rules performed poorly. It is particularly interesting to notice that anti-Hebbian rules have been reported at corticostriatal synapses^32–38,67^, and therefore enable the correct classification of patterns of sequential cortical activity.

These experiments have however pointed out that the anti-Hebbian rules, coupled with non-associative reward-LTP lead to an equilibrium where the neuron oscillates between sub- and supra-threshold states. The accuracy suffers from these dynamics and does not reflect that the network has indeed learned the correct combination of weights to elicit a spike at the end of the pattern. To avoid spurious fluctuations of accuracy due to the structure of learning responses described above, we defined the MaxAccuracy quantification (defined in Material and Methods), and used that measure for most subsequent analyses.

### Anti-Hebbian learning allows the learning of multiple spike sequences

We next tested learning accuracy on a set of more complex tasks (defined in the Material and Methods).

**Fig. 4:**
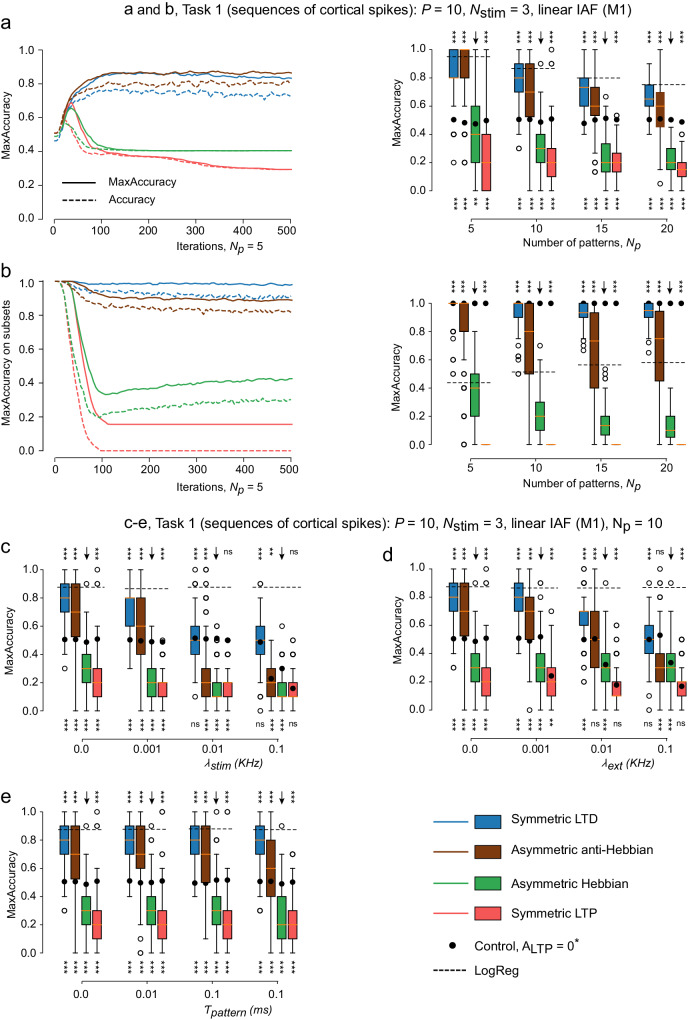
Anti-Hebbian rules and non-associative LTP enable efficient learning with a single linear integrate-and-fire MSN (M1). **a** A-Hebbian STDP supports the learning of sequences of cortical inputs [Task 1] (left) Averaged accuracy (dashed lines) and MaxAccuracy (solid lines) as a function of learning iterations, for different STDP rules, for *N*_*p*_ = 5 patterns. (right) Statistics of the final MaxAccuracy as a function of the number of presented patterns *N*_*p*_, for different STDP rules. **b** Anti-Hebbian rules lead to a specific equilibrium where smaller subsets of rewarded patterns do not lead to a spike of the MSN. (left) [Task 1] Averaged accuracy (dashed lines) and MaxAccuracy (solid lines) as a function of learning iterations, for different STDP rules, when testing subpattern of *N*_*p*_ = 5 learned patterns. (right) Statistics of the final MaxAccuracy when testing subpatterns of *N*_*p*_ learned patterns, for different STDP rules. Influence of different types of noise on learning and performance in Task 1, comparing the final MaxAccuracy upon variation of cortical inputs noise ${\lambda }_{{{{{{{\rm{stim}}}}}}}}$ (**c**), external noise *λ*_ext_ (**d**) and jitter in spike times during pattern presentation *τ*_pattern_ (**e**), for different STDP rules. Training was performed for 500 patterns iterations, with test sessions every *N*_*p*_ iterations. Boxplot descriptions include *N* = 250 simulations. Statistical *t* test from scipy.stats Python library; *: *p* < 0.05, **: *p* < 0.005, ***: *p* < 0.0005. (below) (H0): networks without supervision (*A*_reward_ = 0), compared with networks with supervision (*A*_reward_ = 0.9). (above) (H0): networks with asymmetric Hebbian STDP, compared to other STDP rules.

We started by presenting the network with spatio-temporal sequences of spikes, with a fixed delay between each spike (Task 1). The results for this task, with *P* = 10 cortical neurons, *N*_*p*_ = 5 patterns and ${N}_{{{{{{{\rm{stim}}}}}}}}=3$ maximum spikes per pattern are presented in Fig. 4a. The temporal evolution of the accuracy (dashed lines) and MaxAccuracy (solid lines) are represented for all of the four types of STDP considered (Hebbian or anti-Hebbian, with symmetric or asymmetric polarity). We found that both types of anti-Hebbian learning rules learn to classify correctly the patterns. As previously explained, the synaptic weights converged to a dynamically steady state where the MSN alternated a few correct responses with one wrong response that initialized a new sequence of correct responses. Using the MaxAccuracy quantification, we observed higher levels of performance indicating that the network discriminated patterns correctly. Hebbian rules did not perform well in this task, leading to low accuracies. It is interesting to note that MaxAccuracy and accuracy converged to the same values for Hebbian rules, showing that MaxAccuracy did not always improve the accuracy value.

Similar results were obtained for various numbers of patterns *N*_*p*_ (Fig. 4a). For all tasks, only anti-Hebbian rules performed significantly better than the control condition (reward-LTP absent, black circles in the graphs, with significance levels indicated at the bottom of each condition). Hebbian rules performed significantly worse than without supervision. When comparing with results from the logistic regression, anti-Hebbian rules performed slightly worse than the classical machine learning algorithm. As a conclusion, anti-Hebbian rules enable efficient learning when learning spatio-temporal patterns of spikes, while Hebbian rules perform worse than a non-supervised network.

To further investigate the equilibrium reached by anti-Hebbian rules while memorizing the patterns, we tested the response of the network to randomly selected subpatterns of the rewarded patterns (Fig. 4b). For example, if during the task, the pattern (1, 3) was rewarded, we tested the response of the MSN to the patterns $(1,\,\varnothing )$, $(\varnothing ,\,3)$, where $\varnothing$ means that no spike was presented. We computed the MaxAccuracy of the learning of not firing in response to the subpatterns. Anti-Hebbian rules performed significantly better than Hebbian ones, in a task where classical logistic regression produced fewer correct classifications (Fig. 4b).

To appreciate the robustness of these findings, we tested various numbers of cortical neurons *P*, or numbers of maximum spikes by pattern ${N}_{{{{{{{\rm{stim}}}}}}}}$, and found consistent results (Fig. S3a-b), except for asymmetric anti-Hebbian STDP which performs worse for a higher number of stimulations. We also tested if changes in ${A}_{{{{{{{\rm{post-pre}}}}}}}}$ values led to different dynamics (Fig. S3c), and found a less notable influence compared to our observation of a strong dependence of learning ability on *A*_pre-post_ (that in particular distinguishes Hebbian from anti-Hebbian rules). Finally, we tested different values for *A*_reward_ (Fig. S3d). To elicit learning, we needed *A*_reward_ + *A*_pre-post_ < 0, which was verified when *A*_pre-post_ = − 1, for *A*_reward_ < 1. Moreover, maximal learning was achieved when *A*_reward_ + *A*_pre-post_ was small compared to *A*_reward_. Accordingly, we chose in the sequel *A*_reward_ = 0.9 which satisfied both properties.

In conclusion, anti-Hebbian rules do not only learn to correctly classify rewarded patterns, but they also converge to an equilibrium where subpatterns of cortical activity are not sufficient to trigger spiking at the MSN, so that MSNs expressing an anti-Hebbian STDP learn to spike only if the whole pattern is presented.

### Robustness to noise

To test the robustness of learning to spontaneous activity, we introduced three types of noise in the neuronal network dynamics (Fig. 4c–e),

- random cortical spikes at rate of ${\lambda }_{{{{{{{\rm{stim}}}}}}}}$;
- random MSN spikes at rate *λ*_ext_, implemented thanks to *I*_ext_ spikes;
- random jitter in the spike timings during pattern presentation, with standard deviation *τ*_pattern_, leading to more realistic patterns of cortical activity with heterogeneous timing between cortical stimulations.

MaxAccuracy for *P* = 10, *N*_*p*_ = 5 and ${N}_{{{{{{{\rm{stim}}}}}}}}=3$, varying noise levels were presented in Fig. 4c–e. Interestingly, learning of cortical sequences was robust in the presence of random cortical spikes (Fig. 4c), up to 1 Hz, and that symmetric LTD performed well even with higher noise values. Again, this confirmed that asymmetric anti-Hebbian STDP led to more unstable dynamics, and started failing for lower noise intensities than symmetric LTD. The same conclusions held with random MSN spikes (Fig. 4d). In both symmetric and asymmetric anti-Hebbian STDP cases, adding noise with higher frequency (100 Hz) expectedly prevented learning. Finally, adding jitter in the timings of cortical spikes in the pattern presentation did not prevent anti-Hebbian rules from reaching high accuracy (Fig. 4e). The network was robust to noise, and accordingly in the following experiments, we suppressed all noise processes, to concentrate on the higher bounds of the network’s capacity.

### Spiking latency enhances the network’s performance

Using the previous network, anti-Hebbian rules were able to approach classical machine learning accuracy, but they did not perform as well.

Heuristically, integrate-and-fire models have the drawback of firing instantaneously after the depolarization of the membrane potential. This implies that when presented with overlapping patterns, e.g., patterns *A* = (1) and pattern *B* = (1,  2), the neuron is not able to learn to spike after the end of both patterns because it either spikes in response to pattern *A* only, and therefore fires before the end of pattern *B*, or spikes after pattern *B*, and thus do not spike after pattern *A*. This “impatience” of the neuron described by integrate-and-fire models is a classical shortcoming of the integrate-and-fire model. In reality, MSNs exhibit a spike latency, due to specific voltage-gated potassium conductances delaying the emission of the first spike^51,52,68^.

**Fig. 5:**
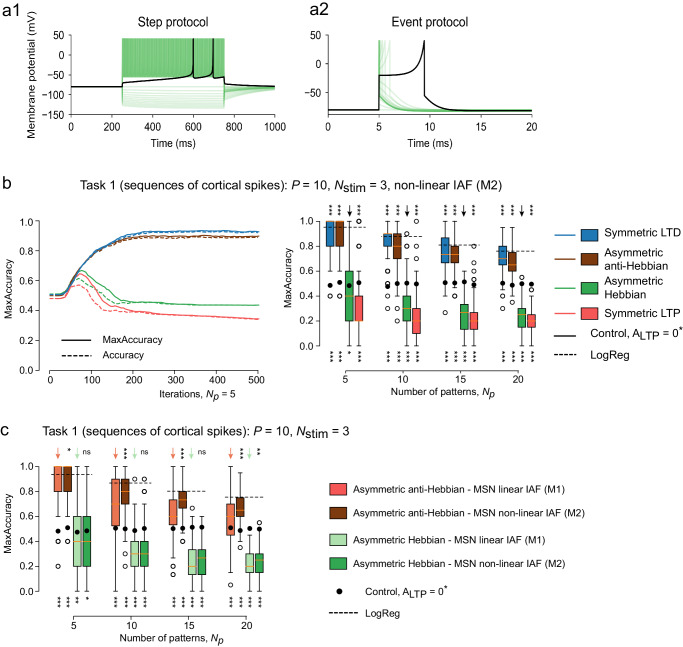
Latency in MSNs enhances the network performance. **a** Response of a non-linear Izhikevich model (M2) to current steps with increasing intensity (**a1**) or presentation of a pulse of current (**a2**). Membrane potential, first spiking event (black). **b** [Task 1] Averaged accuracy (dashed lines) and MaxAccuracy (solid lines) as a function of learning iterations, for different STDP rules, for *N*_*p*_ = 5 patterns for non-linear Izhikevich (M2) model. (right) Statistics of the final MaxAccuracy as a function of the number of presented patterns *N*_*p*_, for different STDP rules. **c** Statistics of the final MaxAccuracy as a function of the number of presented patterns *N*_*p*_, for asymmetric Hebbian and anti-Hebbian STDP, comparing linear IAF (M1) and non-linear Izhikevich (M2) models. Training performed for 500 patterns presentations, with test sessions every *N*_*p*_ iterations. Boxplot descriptions include *N* = 250 simulations. Statistical *t* test from scipy.stats Python library; *: *p* < 0.05, **: *p* < 0.005, ***: *p* < 0.0005. (bottom) (H0): networks without supervision (*A*_reward_ = 0), compared with networks with supervision (*A*_reward_ = 0.9). (**b**, top) (H0): networks with asymmetric Hebbian STDP, compared to other STDP rules. (**c**, top) (H0): (M1, light shade) compared to (M2 dark shade) neuron models, for asymmetric Hebbian STDP (green hue) and asymmetric anti-Hebbian STDP (brown hue).

To test whether the spike latency improves their ability to learn sequences, we modified our neuron model to include a non-linearity and adaptation^53^. We present in Fig. 5a, the MSN membrane potential using the non-linear model (M2), either for step (a1) or pulse (a2) currents. Nonlinear dynamics and spike latency led to dynamics of the membrane potentials closer to electrophysiological data from MSNs recorded in mouse brain slices (compare Fig. S1a1 and Fig. 5a1). Spike latency was particularly notable in the MSN’s response to cortical pulses (Fig. 5a2): when the current pulse was just sufficient to trigger a spike (i.e., equal to the rheobase), initiation of a spike takes several milliseconds.

Figure 5b reports the evolution of MaxAccuracy for learning spatio-temporal patterns (Task 1), using the non-linear neuron (M2). We observed that asymmetric anti-Hebbian STDP performed as well as the logistic regression, which confirmed that the lack of spike latency was responsible for the gap observed between the linear (M1) and non-linear (M2) neurons. We more precisely compared both models and showed that with asymmetric anti-Hebbian STDP, the non-linear neuron (M2) always reached significantly higher accuracies than the linear one (M1) (for *N*_*p* _≥ 10, Fig. 5c).

Anti-Hebbian STDP rules coupled with a latency mechanism for spiking make a simple striatal network as efficient as logistic regression to learn a classification task using biological learning rules.

### Impact of synaptic dynamics on learning

**Fig. 6:**
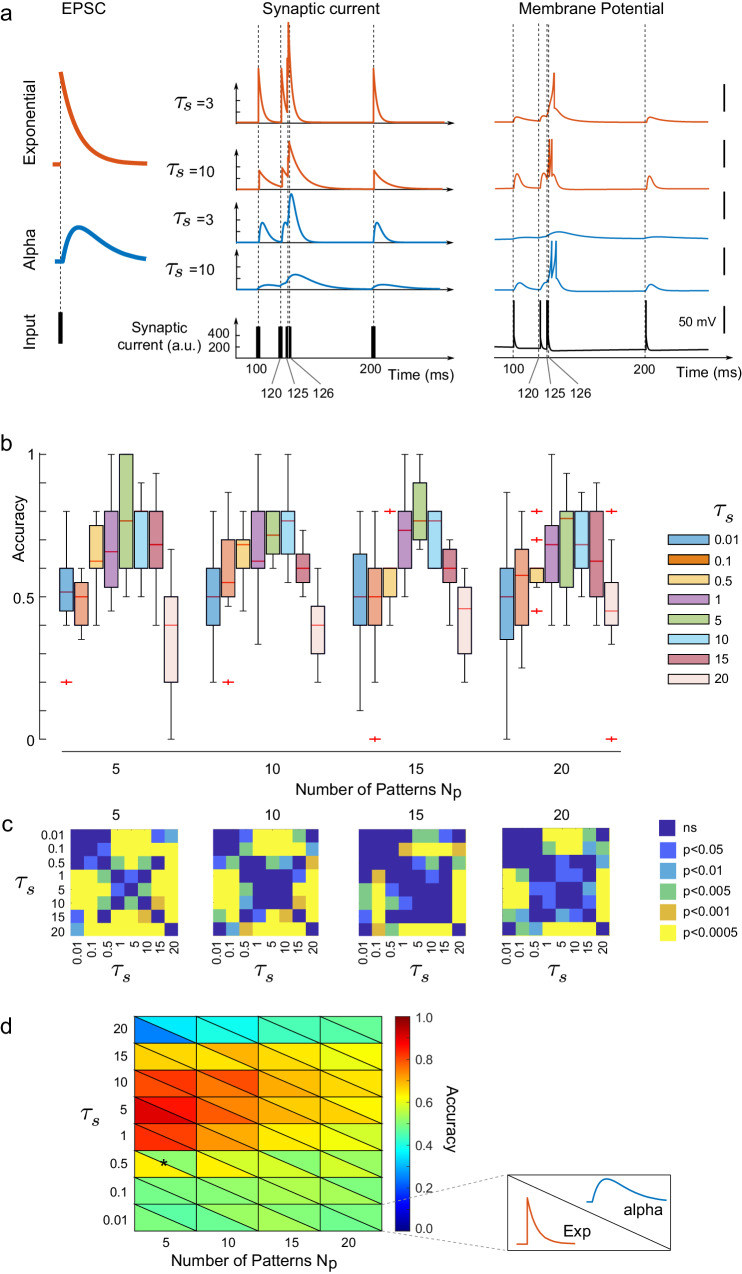
Impact of synaptic timescales on learning. **a** Different synaptic profiles and their impact on MSN firing. Simulations of an exponential (red) or alpha (blue) synaptic current (see equations (2)) in response to a single spike (left) or to an exemplar spike train (five spikes arising at *t* = 100, 120, 125, 126, 200, allowing to appreciate the impact of distant or simultaneous spikes on currents and voltages) for two choices of synaptic timescale *τ*_*s*_ = 3 or *τ*_*s*_ = 10. Left: Voltage of model M2 in response to each of these four currents and for instantaneous synapses (bottom, black trace) for a fixed synaptic weight equal to 6000, arbitrarily chosen to reveal differences between the various situations. Note the difference with Dirac synapses justifying the distinct scaling chosen here compared to Fig. 5. **b** Final accuracy of sequence learning for alpha synapses for eight different synaptic timescales *τ*_*s*_ and four different numbers of patterns to learn, averaged across 40 repetitions of the learning process. We observe an optimal synaptic timescale associated with learning, consistent through the various number of patterns to learn. **c** Statistical significance of the difference between timescales using a two-sample t-test between all pairs of timescales tested. **d** Timescales, rather than synaptic current profiles, control learning: Comparison between learning accuracy between exponential (lower-left hemisquare) or alpha (upper-right hemisquare) as a function of synaptic timescale and number of patterns. Not only is the phenomenology of exponential synapses identical to alpha synapses, but quantitatively learning accuracies end show no significant difference using the two-sample *t* test (*p* > 0.05) except for *τ*_*s*_ = 0.5 and *P* = 5 patterns (two-sample t-test *p* = 0.039), a combination of parameters where exponential synapses are already able to learn sequences well but alpha synapses cannot. Two-sample t-test was used to identify significant differences in (**c**).

A task of sequence classification requires a neuron to resolve finely enough the timing of pre-synaptic spikes, but also ensure enough persistence of the signals to analyze a sequence as a whole. In that view, instantaneous synapses used in the rest of the manuscript provide a maximal time resolution but no persistence of currents, leaving it to the neuron to maintain a trace of the currents received. Biologically, at cortico-striatal synapses, the currents generated by a pre-synaptic neuron are not instantaneous: they display a characteristic continuous time course with a rapid rise and a slower decay, with a timescale on the order of 4–10 ms^69–72^. These are classically modeled by exponential profiles (that neglect the rise time but conserve the typical decay) or more realistic alpha synapses (Material and Methods and Fig. 6a, left)^73^. Contrasting with Dirac synapses inducing an instantaneous change to the neuron’s voltage, continuous post-synaptic currents modify the voltage of a post-synaptic neuron more smoothly as the membrane potential integrates the progressive changes in synaptic current (Fig. 6a, middle). Whether or not the neuron spikes (and when it spikes) in these situations depends both on the parameters of the neurons, synaptic weights and on the profile and timescale of synaptic currents (Fig. 6a, right). Because of this, one may hypothesize that synaptic current profiles have an impact on the learning of sequences.

To test this hypothesis and uncover the way synaptic profiles constrain learning, we computed the learning accuracy of a nonlinear MSN (model M2) with anti-Hebbian STDP and alpha synapses with various timescales. We observed that the learning accuracy showed a non-monotonic dependence on timescale with maximal learning accuracy reached for *τ*_*s*_ = 10 ms (Fig. 6b). This optimal timescale was consistent when we changed the number of patterns presented and maintained a statistically significant difference with biologically too slow (*p* ≥ 15) or too fast (*p* ≤ 0.5) synapses for all choices of numbers of patterns learned (Fig. 6c). Heuristically, the existence of an optimal timescale can be understood from the tension between the necessity to resolve spike times finely and the requirement to maintain spike information throughout the pattern presentation. While the accuracy obtained depends on the scaling of synaptic currents, because the total synaptic current transmitted by all alpha synapses is normalized, this simulation also suggests that synaptic timescales allow for an efficient repartition of the synaptic current in time enabling optimal learning of sequences.

Alpha synapses encompass both a rapid rise time of currents and an exponential decay. To disentangle the respective roles of rise and decay time, we compared the learning accuracy of networks endowed with alpha and exponential synapses with the same timescale (Fig. 6d). Quite strikingly, we found that not only the phenomenology was identical between the two models, but also the accuracies obtained were all statistically consistent with each other (*p* > 0.05) for all choices of timescales and numbers of patterns learned, except for the learning of five patterns with *τ*_*s*_ = 5 where exponential synapses allow better learning of sequences than the alpha synapse. This consistency suggests that the decay timescales, rather than the rise time of synaptic currents, are responsible for the observed phenomenology.

Altogether, this study showed that more realistic profiles of EPSCs yielded a phenomenology consistent with instantaneous synapses and allowed learning sequences, and that distributing the input in time allowed using smaller synaptic currents for learning, with optimal timescales for learning consistent with physiological decay timescales.

### Inhibition in striatal networks improves learning

**Fig. 7:**
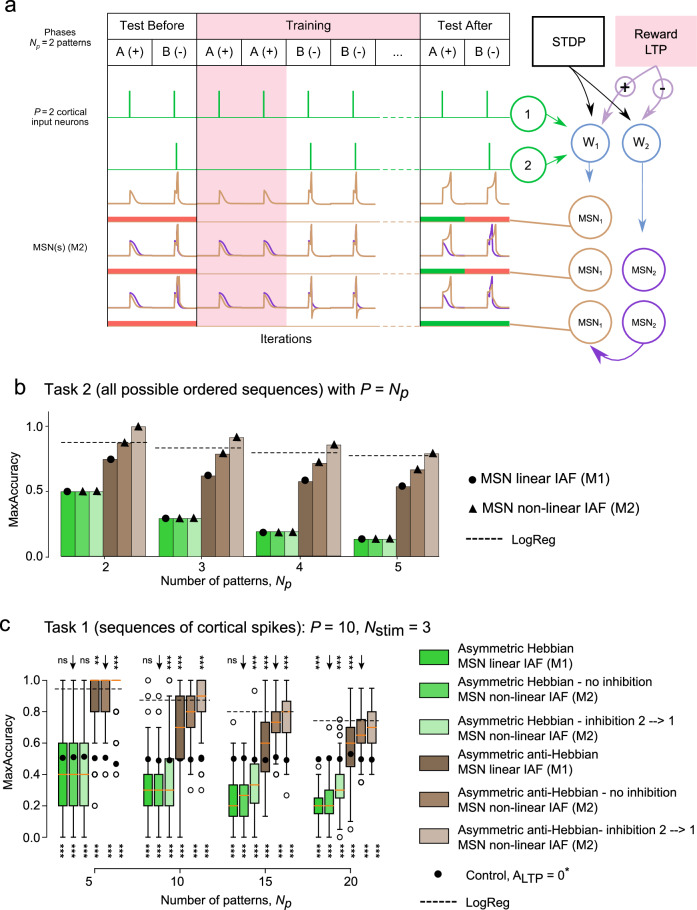
Lateral inhibition facilitates the learning of complex pattern sequences. **a** Lateral inhibition in the striatal network. Schematic representation of the striatal network (right) with *P* = 2 cortical neurons (green), and three different models for MSN activity: (i) one striatal neuron (MSN) modeled as a non-linear integrate-and-fire neuron (M2), (ii) two striatal neurons (MSN_1_ in brown, and MSN_2_ in purple) without collateral inhibition, (iii) two striatal neurons with collateral inhibition from MSN_2_ to MSN_1_. Two mechanisms of synaptic plasticity are considered in the dynamics of the synaptic weight *W* (blue): STDP and LTP related to the reward signal (reward-LTP) (red). Reward-LTP is presented for rewarded patterns at MSN_1_ and for non-rewarded patterns at MSN_2_. Example of the learning task (left), with test sessions and the training protocol (middle). *N*_*p*_ = 2 patterns *A* and *B* are presented to the network, with *A* being rewarded (+) and *B* non-rewarded (−) (for MSN_1_). In the test sessions, below the MSN potential, are represented accuracy results (correct classification in green, wrong classification in red). Spiking activity of the cortical neurons (green) is represented along with the MSN membrane potential *V* of the output neuron(s). **b** Lateral inhibition improves learning all possible patterns for a small number of neurons. [Task 2] MaxAccuracy when learning all possible sequences for *P* neurons, for asymmetric Hebbian and anti-Hebbian STDP, comparing linear integrate-and-fire (M1) and non-linear Izhikevich (M2) models, in the absence (*J* = 0) or presence (*J* = − 0.5) of lateral inhibition. **c** Consequence of lateral inhibition when learning sequences of cortical inputs [Task 1] MaxAccuracy as a function of the number of presented patterns *N*_*p*_, for asymmetric Hebbian and anti-Hebbian STDP, comparing linear integrate-and-fire (M1) and non-linear Izhikevich (M2) models, in the absence (*J* = 0) or presence (*J* = − 0.5) of lateral inhibition. [Task 2] Training performed for 2000 patterns iterations, with test sessions every five iterations. [Task 1] Training performed for 500 patterns iterations, with test sessions every *N*_*p*_ iterations. Boxplot descriptions include *N* = 250 simulations. Statistical *t* test from scipy.stats Python library; *: *p* < 0.05, **: *p* < 0.005, ***: *p* < 0.0005. (below) (H0): networks without supervision (*A*_reward_ = 0), compared with networks with supervision (*A*_reward_ = 0.9). (above) (H0): (M2) neurons without collateral inhibition (*J* = 0) for asymmetric Hebbian STDP (green) and asymmetric anti-Hebbian STDP (brown).

While taking into account non-linearities and adaptation produce latencies that may allow the appropriate learning of nested rewarded patterns, the excitatory nature of the cortico-striatal input prevents the system from learning rewarded pattern *A* and a non-rewarded pattern *B* that contains *A* (Fig. 7a). Indeed, if the MSN spikes for pattern *A*, implying necessarily that it spikes for pattern *B* since it receives even more excitation (Fig. 7a); in contrast, if the neuron does not fire for pattern *B*, then a fortiori the MSN does not receive enough excitation to spike for sub-pattern *A*. We note that a similar issue arises with the logistic regression when we constrain the weights *W* to be positive. Biologically, the striatum is constituted by a large number of MSNs, sharing part of their input, receiving distinct neuromodulation, and interacting together through collateral inhibition^58–62^. Heuristically, this collateral inhibition could provide a mechanism to learn such nested patterns.

We explored the role of collateral inhibition by considering a simple two-neuron network model, where each MSN (MSN_1_ and MSN_2_) was a non-linear neuron (M2), which integrated the same cortical activity through two different weight matrices *W*_1_ and *W*_2_ (Fig. 7a), and MSN_1_ might be inhibited by MSN_2_ through an additional current. We considered that both cortico-striatal synapses underwent the same STDP rules, but opposite rewarding signals.

In the absence of collateral inhibition, MSN_1_ learned to respond to pattern *A*, and as a consequence also spiked for pattern *B* ⊃ *A*, while MSN_2_ spiked after pattern *B*. Inhibition from MSN_2_ induced a strong enough inhibition of MSN_1_ potential to prevent it from spiking, leading to the correct classification of both patterns. Going beyond this specific case, we investigated in detail how, statistically, collateral inhibition impacted accuracies.

To study more extensively this network property, we tested the ability of our models to learn nested patterns (Task 2). The results are presented in Fig. 7b, for *P* = 2, 3, 4 or 5 cortical neurons. cortical neurons *P*. In agreement with the example of Fig. 7a, the network with collateral inhibition correctly classified all sequences of patterns for *P* = 2, and remained close to the optimal performance for higher values of *P*, and in particular, outperformed both the network without inhibition and the logistic regression.

Our choice of rewards for MSN_2_ as a complete opposite of the rewards of MSN_1_ (labeled differential rewards below) is a theoretical situation and therefore unlikely to occur. Generally, MSN_2_ may also be rewarded in response to a pattern rewarded for MSN_1_ or be indifferent. We tested learning accuracy when both MSNs were rewarded or not for the same patterns (same rewards), and found that only the differential rewards scheme was yielding a significant improvement in accuracy (Fig. S4).

We also tested this network when learning spatio-temporal patterns (Task 1, Fig. 7c). Collateral inhibition led to a significantly higher performance for all parameters tested. The conclusions were strongly significant for a small number of patterns *N*_*p*_, but the significance tended to decrease for a higher number of patterns.

These results were confirmed for different sets of *P* neurons (Fig. S5a), various maximum number of spikes by pattern ${N}_{{{{{{{\rm{stim}}}}}}}}$ (Fig. S5b) or different values of collateral inhibition (Fig. S5c).

Overall, asymmetric Hebbian STDP, for both tasks, still leads to poor performances, while asymmetric anti-Hebbian STDP reaches high accuracy. The two biologically documented and relevant MSN properties that have been added, spiking latency and collateral inhibition, both lead, through their specific mechanisms, to significant increases in accuracy.

We finally tested the ability of our different neuronal networks to learn more complex inputs, without having a fixed delay between spikes. We either tested the learning of patterns with jittered delays (Task 3) or Poisson structures (Task 4, Material and Methods).

**Fig. 8:**
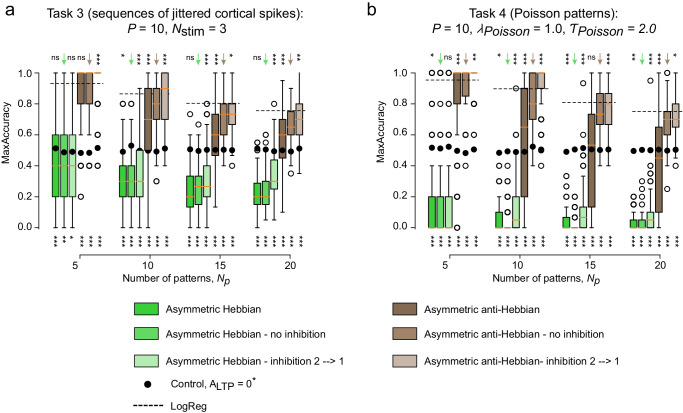
Striatal learning of more realistic patterns. **a** Learning patterns with jittered inputs [Task 3] MaxAccuracy as a function of the number of presented patterns *N*_*p*_, for asymmetric Hebbian and anti-Hebbian STDP, comparing linear integrate-and-fire (M1) and non-linear Izhikevich (M2) models, in the absence (*J* = 0) or presence (*J* = − 0.5) of lateral inhibition. **b** Learning patterns of Poisson spike trains [Task 4] MaxAccuracy as a function of the number of presented patterns *N*_*p*_, for asymmetric Hebbian and anti-Hebbian STDP, comparing linear integrate-and-fire (M1) and non-linear Izhikevich (M2) models, in the absence (*J* = 0) or presence (*J* = − 0.5) of lateral inhibition. Training was performed for 500 pattern iterations, with test sessions every *N*_*p*_ iterations. Boxplot descriptions include *N* = 250 simulations. Statistical *t* test from scipy.stats Python library; *: *p* < 0.05, **: *p* < 0.005, ***: *p* < 0.0005. (below) (H0): networks without supervision (*A*_reward_ = 0), compared with networks with supervision (*A*_reward_ = 0.9). (above) (H0): (M2) neurons without collateral inhibition (*J* = 0) for asymmetric Hebbian STDP (green) and asymmetric anti-Hebbian STDP (brown).

We present the classification results for jittered delay patterns (Task 3, Fig. 8a) and Poisson patterns (Task 4, Fig. 8b and Fig. S6), for different values of number of patterns *N*_*p*_. We observed that even with complex inputs, global performances were consistent with what was observed for spatio-temporal patterns with fixed delays (Task 1), in particular with collateral inhibition leading to higher accuracies than logistic regression. These observations depend on the duration of the pattern, and as expected accuracy decreases, albeit remaining above chance, when considering increasing long patterns (Fig. S6).

## Discussion

The biological mechanisms involved in the learning of sequences are largely elusive and likely multifarious. To lift part of the veil on this complex phenomenon, we developed simple models of corticostriatal networks and explored their ability to learn and identify sequences. Notably, we studied the possible role of the synaptic plasticity observed experimentally at the level of MSNs^28^, spike latency^51,52,68^ and collateral inhibition^58–62^, at the level of one MSN integrating spikes from a population of cortical neurons. We designed a simple learning task as a mock-up of procedural learning to test this ability. In this task, the MSN learns to correctly classify patterns of spikes (precisely timed sequences of cortical spikes) by spiking at the end of the pattern for a specific subset of patterns, and not spiking for others. Our simulation results show that even the simplest striatal network models, endowed with two types of synaptic plasticity, anti-Hebbian learning rules (either symmetric LTD or asymmetric anti-Hebbian STDP) and non-associative reward-LTP, perform well in this task. However, we also observed that some types of combinations of patterns are harder to learn simultaneously by the simplest networks, in particular when learning nested patterns of spikes. This is where we showed that spike latency, a prominent electrophysiological property of MSNs^51,52,68^, solved the problem of early spiking during a sub-pattern, and networks of neurons with spike latency were shown to achieve similar performance as classical logistic regression. However, in that case again, a difficulty arises when learning nested patterns, whereby the full pattern is not rewarded but a subpattern is rewarded. We observed that the addition of a second MSN that learns the reverse associations of patterns and that inhibits the first MSN through lateral inhibition fully solved the problem. In the latter situation, the striatal network in fact outperformed classical algorithms.

Our contribution is thus fourfold. We (i) developed a conceptual framework to test for sequence learning, (ii) showed that anti-Hebbian learning rules as observed in cortico-striatal synapses naturally endow simple models with the ability to learn sequences, (iii) observed that spike latency, as widely evidenced in MSN^51,52,68^, enhances sequence learning and (iv) observed that striatal networks with collateral inhibition^58–62^ further improve learning of sequences that can even outperform artificial algorithms in learning sequence.

Our choice was to use models as simple as possible to specifically identify the impact of individual properties for biological learning rules, MSN spike latency property and lateral inhibition in small networks. Of course, this model does not encompass the full complexity of striatal function. In particular, several recent contributions have questioned the relevance of simple, pair-based STDP rules in learning^15^, in specific contexts. Evolutions of classical Hebbian learning rules including some models encompassing a dependence of LTP and LTD upon voltage and frequency were developed^74^, triplet rules^75^, three-factor learning rules^76^ or even novel paradigms^77^ could be explored. Notwithstanding its shortcomings, pair-based STDP rules are shown here to be sufficient to endow the system with the ability to detect sequences. An interesting avenue would be to explore how more realistic plasticity models, either based on complex patterns^15,74,75,77^ or even more biophysically realistic models of the synapse^78–80^, of neurons^81,82^ or of rewards and neuro-modulation^83,84^ would enhance the ability of networks to learn and if regimes of efficient sequence learning correspond to biophysically realistic parameter ranges. Moreover, the task considered here is too elementary and the number of MSNs too modest to draw any firm conclusion on the physiological function of the cortico-striatal axis. However, this simplicity also comes as an advantage for deciphering the role of a plasticity rule devoid of network effects. The results indeed show that MSNs, mainly because of the anti-Hebbian STDP, can decode a full sequence of cortical/thalamic activity (which is already associative) to extract from this distributed and complex firing a simple command to execute. Of course, this is not a biological claim or limiting in any form, and we expect that the striatum will not be limited to a collection of neurons firing one spike after complete sequences; we in fact expect the striatum to extract other types of information, but the combination of intrinsic and network properties allows the striatum such sequential activity pattern optimized reading. Some biological observations argue in favor of this possibility at cortico/thalamo-striatal synapses where associative inputs have a strong temporal organization. Indeed, such sequential activity patterns were reported in the cerebral cortex and thalamic nuclei in non-human primates and rodents during the completion of behavioral tasks. Striatum receives monosynaptic inputs from the whole cortical areas and from some thalamic nuclei^24,85–88^. The MSNs act as coincidence detectors of distributed patterns of cortical and thalamic activity, because of specific intrinsic properties (mainly due to voltage-gated potassium conductances, *i*_*H*_ and *i*_*R*_) such as a very hyperpolarized resting membrane potential, I-R bell-shape relationship, inward recitifying I–V relationship, delayed first action potential^51,52,68^. Because of these basic intrinsic properties, associated with anti-Hebbian plasticity expression together with the existence of inhibitory collaterals, we argue that MSNs can decode cortico/thalamo-striatal activity patterns organized in temporal sequences.

This work presents a proof of concept that anti-Hebbian STDP allows for the learning of spike sequences. It is deeply rooted in ample biological observations of the presence of sequential activity in the cerebral cortex^2,10,29,46–50^ and in the striatum^7,29–31,89^, and on the anti-Hebbian type of plasticity observed at cortico-striatal synapses in vitro^32–36^ or in vivo^37,38^. In parallel, striatum plays a crucial role in procedural learning ^27,28^. The dorsal striatum, the main input structure of the basal ganglia, receives excitatory inputs from all cortical areas and from thalamic nuclei and has been shown to play a major role in action selection^25–27^ and to be a prominent site for memory formation and procedural learning^28^. In this variety of tasks, it could be expected that the striatum uses information from sequences of evidence to take a decision^27,30,31^. However, the link between these two biological observations was never established and seems difficult to isolate experimentally. And in fact, direct evidence of the implication of STDP on function has been certainly hard to acquire in all domains of plasticity and learning. In the particular domain of procedural and sequence learning, how sequences can be decoded biologically is still unclear. Based on previous findings that anti-Hebbian STDP is expressed in dorsal striatum^36^ and specific properties of MSNs (very hyperpolarized resting membrane potential, I-R bell-shape relationship, delayed first action potential, inhibitory collaterals), our simulations point toward an optimized decoding of sequential activity patterns thank to anti-Hebbian plasticity. Here, we propose a cellular basis for learning sequential cortical and/or thalamic activity patterns in the dorsal striatum thanks to anti-Hebbian plasticity rules. To this day, there is a lack of experimental evidence regarding the occurrence of “natural” cortico/thalamo-striatal STDP-like patterns in behaving animals. This remains to be investigated with for example the rise of the Neuropixel multi-unit in vivo recordings.

Of course, the networks studied are toy models and provide a simplified view of biology. More realistic models, including multiple striatal neurons belonging to various populations emulating for instance DLS and dorsomedial (DMS) striata that have been shown to display distinct types of anti-Hebbian plasticities (symmetric LTD and asymmetric STDP)^36^, multiple pathways, downstream neurons, possible feedback loops and precise neuromodulation systems, will allow assessing whether realistic models also show similar abilities. Incorporating the direct and indirect pathways can also be interesting when considering the influence of striatal plasticity in the process of action selection, which heavily rely on the distinctive dynamics specific to each pathway^90^. A similar dichotomy exists when comparing the DMS and DLS. Both regions are reportedly involved in distinct steps of procedural learning, specifically goal-directed behavior and habits^25–27,91^. Differences in corticostriatal STDP have been shown to exist experimentally, and a similar striatal network was developed in ref. ^36^ to study the influence of the different types of anti-Hebbian STDP on the flexibility and maintenance of learning. Another advantage of these models would be to provide more realistic models of collateral inhibition. Indeed, one particular assumption made in our model of two-cell system with collateral inhibition required us to assume that all the collateral inhibition received by the MSN arose from a single sister cell, which does not capture all the complexity of the network, and required to assume that the sister MSN learned a negative image of the first MSN. This assumption will likely no more be required in larger scale models of the striatum, whereby multiple cells could contribute to the inhibition of different patterns. This type of larger-scale model will also allow investigating the ability of networks to learn multiple tasks and better appreciate the learning capacity of striatal networks, and delineating more finely the respective roles of the different compartments involved in striatal function in the learning of sequences.

Feedforward inhibition, through GABAergic interneurons, was not modeled here, and would likely have a different impact on learning. Cortico-striatal synapses on fast-spiking parvalbumin interneurons display Hebbian plasticity ^92^, which would lead to new behaviors when coupled with anti-Hebbian STDP at MSNs. More generally, the striatum is composed of many micro-circuits, different compartments that display a great variety of STDPs, and a global model of this system, including different inputs (from the cortex and the thalamus), anatomo-functional compartments of the striatum (DMS/DLS) and types of neurons (MSNs, GABAergic interneurons, dopaminergic neurons, cholinergic neurons) could lead to a general theory of striatal learning.

The reward signaling used in the present model was restricted to simple supervision through the potentiation of synaptic weights associated with presynaptic spikes during rewarded patterns. Detailed models, in particular three-factor learning rules^93–95^ could also be used in this context, and in particular by proposing more realistic models of how rewards can be implemented through, e.g., dopaminergic signaling, for instance along the lines of the model of ^57^ developed for Hebbian STDP.

Such models require to identify triggers of dopamine. Such models could further integrate the fact that dopaminergic neurons are not only modulated by the value of rewards (or of the reward-prediction error). Indeed, dopaminergic neurons of the substantia nigra pars-compacta, which are responsible for dopamine in the dorsal striatum, are known to be also directly stimulated by MSNs originating from striosomes^96^. The integration of the dopaminergic circuit could therefore lead to more realistic studies of the influence of reward on learning in the striatum.

Altogether, this study provides another view into the functional role of anti-Hebbian STDP in the striatum in relationship with episodic memory and learning of temporal sequences. This STDP rule is fully pairwise and thus allows for efficient algorithmic implementations. It is quite remarkable to note that other artificial models for learning temporal sequences have used anti-Hebbian STDP^16^. As observed here, this ability to learn sequences seems to be in the very nature of the anti-Hebbian learning. When learning sequences, the network is required to sit on a narrow region where it develops the ability to spike in response to a sequence, which in turn alters its ability to further spike in response to this same sequence. Such learning rules appear to allow the network to self-organize near this critical transition between spiking and non-spiking^97^. (Self-organized) critical systems are notorious for showing rich properties^98,99^; it is likely that these phenomena also shape the distribution of synaptic weights according to some prescribed input statistics or patterns, the study of which would be an interesting question that could be also compared to data. Anti-Hebbian spiking networks with LTD provide an example of self-organization to criticality, the theoretical study of which constitutes a potentially rich and fascinating perspective of this work.

## Materials and methods

We used two integrate-and-fire models to represent the MSN dynamics, a simple leaky integrate-and-fire model (M1) and a slightly more realistic adaptive nonlinear model (M2).

### Leaky integrate-and-fire model of the MSN (model M1)

For model M1, the MSN was modeled as a linear leaky integrate-and-fire neuron^56,73,100^. In this model, the voltage of the MSN evolves according to a linear equation as its cortical and external inputs, and fires when the voltage exceeds a fixed threshold. In detail, between two spikes, the membrane potential *V* of the neuron satisfied a linear differential equation:

1 $$\tau \frac{dV}{dt}=-(V(t)-{V}_{{{{{{{\rm{eq}}}}}}}})+RI(t)+\sqrt{\tau }{V}_{{{{{{{\rm{noise}}}}}}}}(t)$$

Spikes were emitted when the voltage exceeded a threshold *V*_th_, at which time the MSN’s voltage was instantaneously reset to *V*_r_ and resumed input integration after a delay *τ*_refractory_ = 10 ms. Noise and neural activity external to the considered network were summarized in a Gaussian white noise term *V*_noise_(*t*) with standard deviation *η*_noise_ = 0.5 mV.

To fit the model, we used electrophysiological recordings of MSNs (*n* = 16) performed in acute brain slices of the dorsolateral striatum (DLS) (Fig. S1a1, data collected by Elodie Perrin in Venance’s lab). Whole-cell patch-clamp recordings were performed in acute horizontal brain slices containing the DLS as previously described^34,36^. Briefly, borosilicate glass pipettes of 6–8 MΩ resistance were filled with (in mM): 122 K-gluconate, 13 KCl, 10 HEPES, 10 phosphocreatine, 4 Mg-ATP, 0.3 Na-GTP, 0.3 EGTA (adjusted to pH 7.35 with KOH). The composition of the extracellular solution was (mM): 125 NaCl, 2.5 KCl, 25 glucose, 25 NaHCO_3_, 1.25 NaH2PO_4_, 2 CaCl_2_, 1 MgCl_2_, 10 μM pyruvic acid through which 95% O_2_ and 5% CO_2_ was bubbled. Signals were amplified using EPC10-2 amplifiers (HEKA Elektronik, Lambrecht, Germany). Recordings were performed at 34 ^∘^C and signals were sampled at 10 kHz, using the Patchmaster v2 × 32 program (HEKA Elektronik). All experiments were performed in accordance with the guidelines of the local animal welfare committee and the EU (directive 2010/63/EU).

We extracted from each of these recordings the resting membrane potential *V*_eq_, the spike threshold *V*_th_ and the reset volage *V*_r_. The the change in membrane potential as a function of input current intensity (*I*–*V* curve) was used to estimate the parameter *R* by fitting a linear curve to the experimental data (Fig. S1a2). The timescale parameter *τ* was fitted directly on the electrophysiological traces. The model obtained was able to accurately reproduce spikes and the *I*–*V* curves used for the fit (Fig. S1a 3), but we noted that it did not scale properly for high input currents and did not accurately reproduce the variation of firing rates as a function of input intensity (*F*–*I* curve, Fig. S1a 4).

Each of the 16 MSNs recorded experimentally provided us with a fitted *V*_eq_, *V*_th_, *V*_r_, *R* and *τ* and *C* = *τ*/*R*. These quantities are represented in Fig. S1b, along with their averaged value and, for comparison, the values reported in previous studies (ref. ^56^ for a leaky integrate-and-fire neuron and ref. ^53^ for a non-linear model). The values inferred from experimental data were consistent with canonical models, except for the reset potential *V*_r_ which has sometimes been taken as equal to the resting membrane potential *V*_eq_ (e.g., in ref. ^53^) but was allowed to be different in our model of MSN to account for the fact that electrophysiological traces display reset potentials that are more depolarized than the resting potential, a phenomenon possibility associated with higher excitability immediately after a spike.

### Cortical inputs

The cortical input received by the MSN (term *I*(*t*) in equation (1)) was modeled as the superposition of spikes received from *P* cortical neurons (noted ${I}_{{{{{{{\rm{stim}}}}}}}}(t)$) and a Poisson input (*I*_ext_(*t*)) with rate noted *λ*_ext_:

$$I(t)={I}_{{{{{{{\rm{stim}}}}}}}}(t)+{I}_{{{{{{{\rm{ext}}}}}}}}(t).$$

Unless specified otherwise, each spike induces an instantaneous jump in the MSN membrane potential, with a constant amplitude *W*_ext_ = 1 *nA* for external spikes and synaptic weights $W(t)={({W}_{i}(t))}_{1\le i\le P}$ for each of the *P* cortical neuron considered, that varied through plasticity mechanisms.

$${I}_{{{{{{{\rm{stim}}}}}}}}(t)=	\tau \mathop{\sum}_{1\le i\le P}\mathop{\sum}_{{t}_{i}^{k}\le t}{W}_{i}({t}_{i}^{k}-)\varphi (t-{t}_{i}^{k}),\\ {I}_{{{{{{{\rm{ext}}}}}}}}(t)=	\tau {W}_{{{{{{{\rm{ext}}}}}}}}\mathop{\sum}_{{t}_{{{{{{{\rm{ext}}}}}}}}^{k}\le t}\varphi (t-{t}_{{{{{{{\rm{ext}}}}}}}}^{k}),$$

where we noted, for a function *f* being potentially discontinuous at time *t*, *f*(*t*−) the value reached immediately before the jump, ${({t}_{i}^{k})}_{k\ge 0}$ is the sequence of spikes of neuron *i* and ${({t}_{{{{{{{\rm{ext}}}}}}}}^{k})}_{k\ge 0}$ the sequence of external spike times, that have exponentially distributed inter-spike intervals, and *φ* a Dirac impulse function. The factor *τ* allowed appropriate scaling of weights for direct comparison with experimentally measured excitatory post-synaptic currents (EPSCs).

When testing the role of synaptic timescale on learning (Fig. 6), we considered more realistic EPSC with exponential or alpha profiles:

2 $$\varphi (t)=\left\{\begin{array}{ll}\frac{1}{{T}_{s}}\,{e}^{-\frac{t}{{T}_{s}}}\hfill &{{{{{{\rm{(exponential}}}}} \, {{{{\rm{synapses)}}}}}}}\\ \frac{1}{{T}_{s}^{2}}\,t\,{e}^{-\frac{t}{{T}_{s}}}\quad &\,{{\mbox{(alpha synapses)}}}\,\end{array}\right.$$

where *T*_*s*_ is the synaptic time constant and the normalization coefficients chosen to normalize the synaptic currents (this coefficient ensures that the integrated current is equal to 1 for any *T*_*s*_ chosen). These functions are represented in Fig. 6a together with the superposition of these currents in response to one given spike train.

Our model did not differentiate direct and indirect trans-striatal pathways MSNs for two reasons: (i) we did not aim to model post-striatal processing, and (ii) did not consider the difference between both types of MSNs in their excitability which, considering the simple models of neurons used, would not have led to any difference in performance between both populations, but only to a different scaling of the synaptic weights.

### Non-linear integrate-and-fire neuron model (M2)

We also considered a non-linear neuron model (labeled model M2) introduced in ref. ^53^, where the the voltage *V* is coupled to an adaptation variable *U* through the equations:

$$\left\{\begin{array}{l}C\frac{dV}{dt}=k(V(t)-{V}_{{{{{{{\rm{c}}}}}}}})(V(t)-{V}_{{{{{{{\rm{eq}}}}}}}})-U(t)+I(t)\quad \\ \frac{dU}{dt}=a\left(b(V(t)-{V}_{{{{{{{\rm{eq}}}}}}}})-U(t)\right).\hfill \end{array}\right.$$

In this model, the voltage typically has sharp excursions (and divergences in finite-time), and spikes were considered to be emitted when the voltage exceeds a threshold *V*_th_. At these times, the neuron’s voltage was instantaneously reset to *V*_r_, and the adaptation variable is updated to *U*(*t*−) → *U*(*t*−) + *d*. These models are very versatile depending on the parameter set^101,102^. They were used here in a regime that produces dynamics comparable to MSN, as proposed in ref. ^53^ (Table 1). The parameters were compared to the integrate-and-fire model (M1) ones in Fig. S1b. To appropriately scale the currents in the case of Dirac impulses, the currents ${I}_{{{{{{{\rm{stim}}}}}}}}$ and *I*_ext_ defined above are multiplied by *RC*, with *R* a scaling factor set as *R* = 100 MΩ.

### Two-neuron network with inhibition

When considering a system composed of two neurons, we modeled each MSN (MSN_1_ and MSN_2_) using the non-linear integrate-and-fire model M2 and assumed that they integrated the same cortical activity through two different weight matrices *W*_1_ and *W*_2_ (Fig. 7a). In addition to this input, MSN_1_ was inhibited by MSN_2_ through an additional current:

$${I}_{2}(t)=RCJ\mathop{\sum}_{{t}_{{{{{{{\rm{MS{N}}}}}}_{2}}}}^{k}\le t}\delta (t-{t}_{{{{{{{\rm{MS{N}}}}}}_{2}}}}^{k}),$$

where $({t}_{{{{{{{\rm{MS{N}}}}}}_{2}}}}^{k})$ were the spike times of MSN_2_, and *J* = − 0.5 *nA* (control with no inhibition corresponded to *J* = 0). Rewards differed between the two neurons, which were assumed to learn opposite tasks (i.e., MSN_2_ was rewarded for non-rewarded patterns of MSN_1_, Fig. 7a). The accuracy was read out on MSN_1_ only.

### Corticostriatal synaptic plasticity

Synaptic weights from the *P* cortical neurons to the MSN were subject to pair-based STDP^14^, modeled as synaptic weight updates arising after each spike according to the spike timing relative to all previous spikes of the other neuron (all-to-all implementation in the parlance of ref. ^103^). In detail:

- If the MSN spiked at time *t*_post_ (post-synaptic spike), then all weights were updated. Noting ${t}_{{{{{{{\rm{pre}}}}}}},i}$ the previous spikes of cortical neuron *i*, the synaptic weight *W*_*i*_ was updated according to:
  $${W}_{i}({t}_{{{{{{{\rm{post}}}}}}}})={W}_{i}({t}_{{{{{{{\rm{post}}}}}}}}-)+\varepsilon \mathop{\sum}_{{t}_{{{{{{{\rm{pre}}}}}}},i} < {t}_{{{{{{{\rm{post}}}}}}}}}\Phi ({t}_{{{{{{{\rm{post}}}}}}}}-{t}_{{{{{{{\rm{pre}}}}}}},i})$$
  where *ε* denotes the plasticity rate, chosen in our simulations as *ε* = 0.02.
- If presynaptic cortical neuron *i* ∈ {1, ⋯  , *P*} spiked at time ${t}_{{{{{{{\rm{pre}}}}}}},i}$, noting *t*_post_ the times of the MSN spikes, then the synaptic weight *W*_*i*_ was updated as:
  $${W}_{i}({t}_{{{{{{{\rm{pre}}}}}}},i})={W}_{i}({t}_{{{{{{{\rm{pre}}}}}}},i}-)+\varepsilon \mathop{\sum}_{{t}_{{{{{{{\rm{post}}}}}}}} < {t}_{{{{{{{\rm{pre}}}}}}},i}}\Phi ({t}_{{{{{{{\rm{post}}}}}}}}-{t}_{{{{{{{\rm{pre}}}}}}},i}).$$

Denoting $\Delta t={t}_{{{{{{{\rm{post}}}}}}}}-{t}_{{{{{{{\rm{pre}}}}}}}}$ the timing between the presynaptic spike and the post-synaptic spike, we used an exponential STDP kernel^103^:

$$\Phi (\Delta t)=\left\{\begin{array}{l}{A}_{{{{{{{\rm{post-pre}}}}}}}}\exp \left(\frac{\Delta t}{{\tau }_{s}}\right)\,{{\mbox{if}}}\,\Delta t \, < \, 0\hfill \\ {A}_{{{{{{{\rm{pre-post}}}}}}}}\exp \left(-\frac{\Delta t}{{\tau }_{s}}\right)\,{{\mbox{if}}}\,\Delta t \, > \, 0\quad \end{array}\right.$$

with *τ*_*s*_ = 20 ms (Fig. 1a).

Anti-Hebbian plasticity (i.e., plasticity with post-pre LTP and pre-post LTD, and plasticity with post-pre and pre-post LTD) and the prominence of LTD associated was often combined with non-associative LTP to prevent neurons from becoming silent altogether^42,104,105^. Indeed, synaptic weights involved in the post-synaptic firing are reduced by anti-Hebbian STDP, leading to their decrease, a process that may persist until spike extinction. Beyond this practical observation, non-associative rewards rely on the notion of temporal credit-assignment problem (or distal reward problem) that has ample biological support. In the central nervous system, it has been reported various complex relationships and effects between reward (dopamine) and STDP polarity and shapes depending on dopamine concentration, dopaminergic subtype receptor activated (D1-like vs D2-like), temporality of dopamine delivery relative to STDP induction and also dopamine interactions with other neuromodulators such as acetylcholine^94^. We thus opted to decorrelate reward and STDP to be as generic as possible without favoring any complex interaction. The temporal credit-assignment problem questions the temporal link between the reward and the preceding action to allow reinforcement learning^106^. The temporal credit-assignment problem can be solved with the notion of eligibility trace, a synaptic tag induced by learning that can be transformed into synaptic plasticity by the retroactive effect of various neuromodulators, such as dopamine. Importantly, the eligibility trace allows keeping a synaptic trace from the learning sequence but does not induce synaptic plasticity unless the reward occurs before the extinction of the eligibility trace. Experimentally, this has been demonstrated for example by monitoring the structural plasticity, which was shown to occur when dopamine striatal was released up to 2 s after STDP^107^. In these papers, no evidence of a gradual dependence on the timing of the induced plasticity was reported, so we did not assume any.

We chose non-associative LTP to model reward signals, leading to the following synaptic update rule, where at each presynaptic spike of cortical neuron *i*, the associated synaptic weight *W*_*i*_ was updated by,

$$\Delta {W}_{i}=\varepsilon \,{A}_{{{{{{{\rm{reward}}}}}}}}.$$

where *A*_reward_ is either null (absence of LTP) or positive (non-associative LTP of active presynaptic neurons).

Synaptic weights were clipped within a realist range [*w*_min_, *w*_max_] = [0., 2.] nA. An example of simple synaptic weight dynamics with *P* = 2 cortical neurons, with and without reward is presented in Fig. 1b.

### Pattern recognition in the striatum

Striatal learning is based on the detection of correlated sequences of cortical inputs^29–31^. We emulated learning through different tasks, where *N*_*p*_ spiking patterns of cortical activity were presented to the MSN.

Patterns represent a sequence of cortical activity with duration *t*_duration_ = 50 ms, and combined (i) a specific spatio-temporal pattern of activity involving a subset of cortical neurons (always present at each presentation of the pattern) and (ii) random spiking activity from all cortical neurons.

In Fig. 1c, a simple learning task is detailed with two patterns: pattern *A* which corresponds to a spike from cortical neuron 4 at time *t*_offset_; pattern *B* where cortical neuron 1 spikes at *t*_offset_, followed after a delay *t*_delay_ by a spike of cortical neuron 3.

During learning, the network was presented with patterns, chosen randomly from the set of *N*_*p*_ patterns. Among the patterns, a fixed subset was chosen to be rewarded with a probability of 1/2. Accordingly, the other remaining patterns were defined as non-rewarded patterns. In the example illustrated in Fig. 1c, pattern *A* was chosen to not be rewarded (−) and pattern *B* was rewarded (+). During training, rewarded patterns were subject to a positive potentiation signal (*A*_reward_ > 0) while non-rewarded patterns did not (*A*_reward_ = 0). For all patterns, STDP rules were also applied at the synaptic weight matrix *W* depending on pre- and post-synaptic spikes.

### Learning accuracy quantification

The accuracy of the learning process was estimated through the averaged numbers of correct responses:

3 $${{{{{{\rm{Accuracy}}}}}}}=\frac{1}{{N}_{p}}\mathop{\sum}_{1\le k\le {N}_{p}}{r}_{k}{\sigma }_{k}+(1-{r}_{k})(1-{\nu }_{k}),$$

where *r*_*k*_ = 1 if *k* was a rewarded pattern and 0 otherwise, *σ*_*k*_ = 1 if the MSN spiked after the correlated cortical activity and 0 otherwise, and *ν*_*k*_ = 1 if the neuron spiked, and 0 otherwise.

To correctly classify a rewarded pattern, the MSN should not spike during the cortical pattern but only be elicited after the end of the sequence, to model the capacity of the striatum to make decisions based on whole sequences of cortical activity, and not only on the first spikes.

To avoid spurious fluctuations of accuracy due to the structure of learning responses (namely, unavoidable alternations of successes and failures as studied in Fig. 3) and thus to evaluate accurate learning of sequences, we also defined:

$$\,{{\mbox{MaxAccuracy}}}\,(t)={\max }_{[t-{T}_{1},t+{T}_{1}]}\left\{\,{{\mbox{Accuracy}}}\,(t)\right\}$$

where Accuracy(*t*) represented the value of accuracy computed at time *t*, following Eq. (3), and [*t* − *T*_1_, *t* + *T*_1_] represented an interval of pattern iterations (*T*_1_ taken as 10 test iterations).

For each set of parameters simulations were performed with,

- *A*_reward_ = 0 for all patterns, which served as a control task where no supervision was given to the network to distinguish rewarded patterns.
- *A*_reward_ = 0.9 for rewarded patterns and *A*_reward_ = 0 for non-rewarded ones, to emulate supervised learning using the rewarding signal.

### Algorithm benchmark

Accuracy and MaxAccuracy were computed for both systems and compared to the classification accuracy of more classical algorithms. In detail, we defined an equivalent optimization problem, where the correct classification was learned using logistic regression, implemented with the *lmfit* package. We trained the network by taking as inputs a binary version of the *P* × *N*_*p*_ matrix (*M*_*p*,*n*_), with *m*_*p*,*n*_ = 1 if cortical neuron *n* was spiking during pattern *p*, and *m*_*p*,*n*_ = 0 if neuron *n* did not spike during pattern *p*. The linear matrix in the logistic regression *W* was constrained to only have positive coefficients, thus enforcing the constraints associated with learning in excitatory networks. Comparisons of results obtained using our task with other algorithms from sequential learning appeared not feasible. Indeed, either these algorithms aim at reproducing a target spike train (e.g., Chronotron^20^), and therefore integrate the target into their update rules, or they classify patterns (e.g., Tempotron^21^) without any constraints on the timing of the output spike trains. Our task differed both in what is given in the update rules and the conditions of classification, rendering most comparisons irrelevant. Logistic regression, with positive weights, provides us with a simple way to efficiently compare our task to a baseline of interest.

### Learning tasks

We define four tasks to characterize various dimensions of the learning ability of the network.

Task 1: Learning spatio-temporal sequences of cortical spikes with a fixed delay. Patterns were constructed randomly as follows:

- The number *n* of cortical spikes involved in the pattern was chosen uniformly at random between 1 and ${N}_{{{{{{{\rm{stim}}}}}}}}$.
- The ordered identity of neurons involved in a pattern was chosen uniformly at random among ordered sets of *n* neurons (without replacement) in {1, *P*}.
- The temporal sequence was defined with the first spike at time *t*_offset_, and the following ones presented with a fixed delay *t*_delay_ = 1 ms.
- Finally, each pattern was chosen to be rewarded with probability 1/2.

Task 2: Learning nested sequences of spikes: We tested the ability of the network to discriminate a full sequence of *P* nested patterns, (1), (1,  2), ..., (1,  2,  . . . ,  *P*), when considering all possible combinations of rewarded/non-rewarded patterns (2^*P*^ situations). For example, for *P* = 2, the network was tested on 4 different sets of 2 patterns, (1) and (1, 2), with each pattern being either rewarded (+) or non-rewarded (−) (as illustrated in Fig. 1 c). For this task only, we chose a delay between spikes of *t*_delay_ = 0.5 ms.

Tasks 3: Robustness to noise This task considers patterns formed as in Task 1 but where the times of the spikes within the spatio-temporal pattern are shifted by a uniform random variable (jitter).

Tasks 4: Poisson patterns Patterns learned in Task 4 are generated we considered patterns of cortical activity defined through Poisson processes of intensity *λ*_poisson_ = 1 kHz, on a duration *t*_poisson_ = 2 ms, conditioned to have at least two spikes.

### Statistics and reproducibility

The learning accuracy and other network properties were estimated on a fixed network with synaptic weights frozen (i.e., devoid of plasticity) and in the absence of noise. All patterns were presented, responses were recorded for each pattern presentation, and the MSN membrane potential reset to its resting value between each pattern.

Simulations were performed on a custom code developed in Python 3.X, using the Anaconda suite (Anaconda Software Distribution, Computer software Version 2-2.4.0. Anaconda, Nov. 2016. Web. https://anaconda.com.) and the numeric calculus *numpy* and plotting *matplotlib* libraries. All the code to generate the figures is freely accessible at https://github.com/Touboul-Lab/SequenceLearning. Running the code will allow reproducing the results (with possibly a different random seed). Simulations were run on the INRIA CLEPS cluster and HPC resources from GENCI-IDRIS (Grant 2022-A0100612385), using GNU parallel (Tange, O. (2020, May 22). GNU Parallel 20200522 (“Kraftwerk”), accessed from Zenodo at the link 10.5281/zenodo.3841377). We used an Euler scheme to simulate our network and Poisson processes, with a fixed time-step *dt* = 0.1 ms. An independent code was developed in Matlab to confirm some of the results associated with model M1 in simple situations. It was in particular used to generate Fig. 3.

We used the statistical t-test from scipy.stats Python library or the Matlab ttest2 function (*p < 0.05, **p < 0.005, ***p < 0.0005). Box plots were generated by matplotlib. They represent a box containing the first and third quartiles of the data with a line at the median and whiskers extending from the box to the farthest data point lying within 1.5 times the inter-quartile range (i.e., the length of the box). Flier points are those past the end of the whiskers.

### Reporting summary

Further information on research design is available in the Nature Portfolio Reporting Summary linked to this article.

## Supplementary information

Supplementary Information

Reporting Summary

## Data Availability

All data used to produce the figures was generated via numerical simulations of the freely available code. The extensive data generated by these codes that we used to produce Figures 2, 4–8 as well as Supplementary Figs. S3–S6 is available on Figshare at the link 10.6084/m9.figshare.25355848.v1. Figures 3 and S2 are generated directly by running the associated code.

## Code availability

The code used in this paper is freely accessible at https://github.com/Touboul-Lab/SequenceLearning (10.5281/zenodo.10794811). It contains Python and Matlab code. Python code was developed in Python 3.X, using the Anaconda suite (Anaconda Software Distribution, Computer software Version 2-2.4.0. Anaconda, Nov. 2016. Web. https://anaconda.com.) and the numeric calculus *numpy* and plotting *matplotlib* libraries. Matlab code was developed on Matlab version R2021a.
