# Supplementary material for: Anti-Hebbian plasticity drives sequence learning in striatum
Source: Commun Biol. 2024 May 9;7:555. doi: 10.1038/s42003-024-06203-8 (PMC11082161; doi:10.1038/s42003-024-06203-8)
Supplement: Supplementary file 1 — Supplementary Information [file 42003_2024_6203_MOESM1_ESM.pdf]

Anti-Hebbian plasticity drives sequence learning in striatum - Supplementary Figures

Gaëtan Vignoud[1], Laurent Venance[1,*], and Jonathan David Touboul[2,*]

[1]Center for Interdisciplinary Research in Biology (CIRB), College de France, CNRS, INSERM, Université PSL, Paris, France.
[2]Department of Mathematics and Volen National Center for Complex Systems, Brandeis University, Waltham, MA, USA.
[*]These authors jointly supervised this work.

April 15, 2024

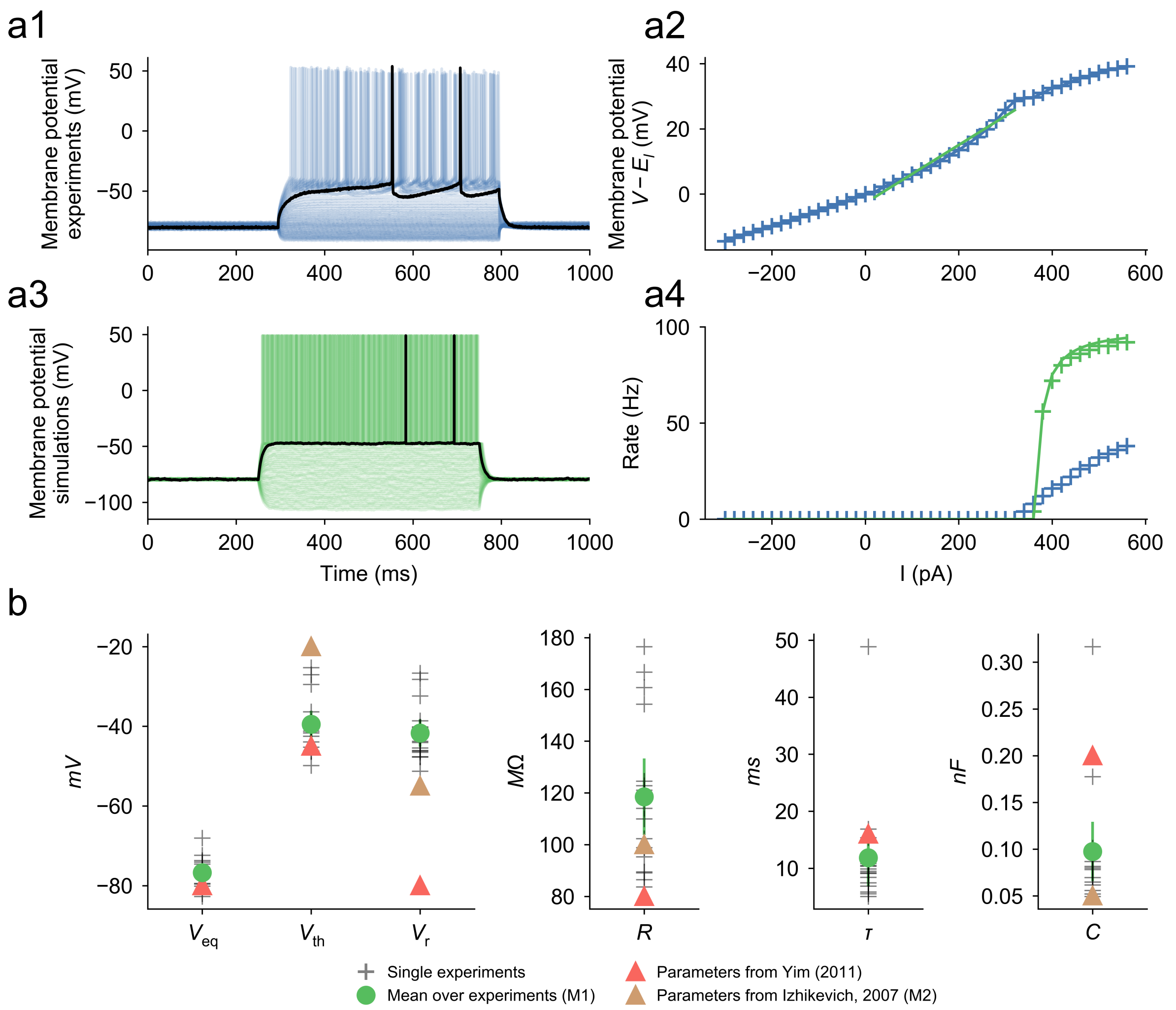

Figure S1: **Integrate-and-fire (M1) model of the MSN.** (a1) MSN electrophysiological data, recorded during an action potential protocol (presentation of current steps with increasing intensity). Membrane potential in mV, first spiking event (black). (a2) mean membrane potential as a function of current intensity (blue). Linear regression was used to determine R (green). (a3) Response of an integrate-and-fire model, using values fitted on experimental data, to an AP protocol (current steps with increasing intensity). Membrane potential, first spiking event (black). (a4) Firing rates as a function of current intensity, experimental data (blue), simulations with an integrate-and-fire neuron (green circle), and exact F-I curve for the integrate-and-fire model (green). Note the deviation of firing rates at high currents, showing that the simple integrate-and-fire model deviates from biological behaviors in these regimes of constant input (all simulations remain away from these levels of DC input where the two behaviors differ).
(b) Integrate-and-fire parameters, from experiments and previous models. Values for the integrate-and-fire model parameters, fitted to each electrophysiological experiment (black crosses). Mean values over all experiments (green circles, M1). Values from the integrate-and-fire parameters from [1] (red triangles) and from Izhikevich models [2] (brown triangles, M2).

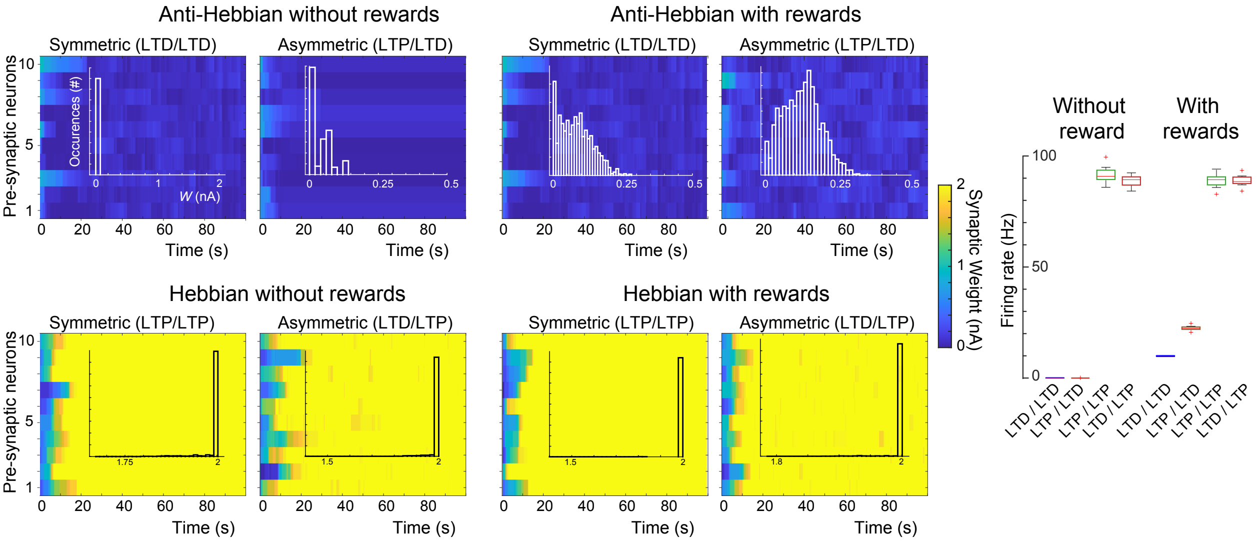

Figure S2: **Synaptic weights and firing rates in response to 10Hz pre-synaptic Poisson input.** (Left) Dynamics of the synaptic weights (heatmap) and stationary distribution (overlaid histogram) for $P = 10$ pre-synaptic neurons 10Hz Poisson firing, in the case of Hebbian or anti-Hebbian symmetric or asymmetric STDP, with or without rewards (stationary distribution computed as the histogram of synaptic weights from time 50s to 100s combining all neurons). (Right) Output firing rate of the MSN (see analogous quantifications for 100Hz input activity in Figure 2a). The simulation duration was adjusted to reflect the (slower) convergence of the weights to a stationary distribution for all types of plasticity considered. Anti-Hebbian rules led to low weights and moderate output firing rates sensitive to the presence of reward-LTP (two-sample t-test between any anti-Hebbian firing rate and any of the other conditions showed significance, with p<0.1 between symmetric LTD and asymmetric STDP without rewards, and all other pairs were associated with p-values below 0.0005), while Hebbian rules showed saturation of the synaptic weights with high firing rates and no sensitivity to the type of plasticity or the possible presence of reward-LTP (p>0.1).

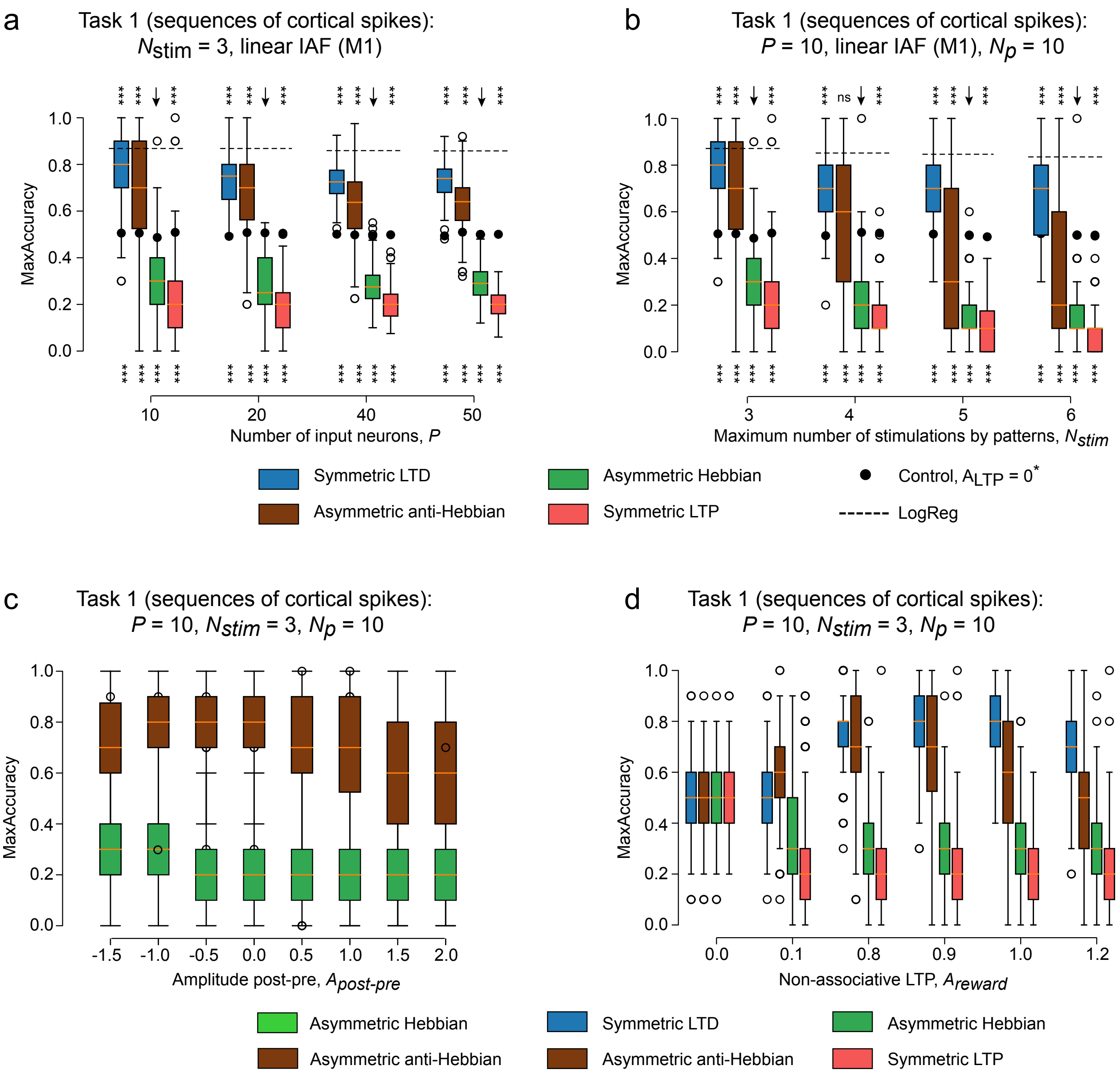

Figure S3: **Influence of parameters on learning with linear IAF model (M1).** [Task 1] (a) MaxAccuracy for different number of cortical neurons P, with N_p=P. Training done for [500, 1000, 2000, 2000] patterns iterations for P=[10, 20, 40, 50], with test sessions every N_p=P iterations. (b) MaxAccuracy for different numbers of cortical stimulations N_{stim}. (c) MaxAccuracy for different values of post-pre amplitude $A_{\text{post-pre}}$. (d) MaxAccuracy for different values of reward-LTP A_{reward}. (statistics and other parameters identical as in Figure 4.)

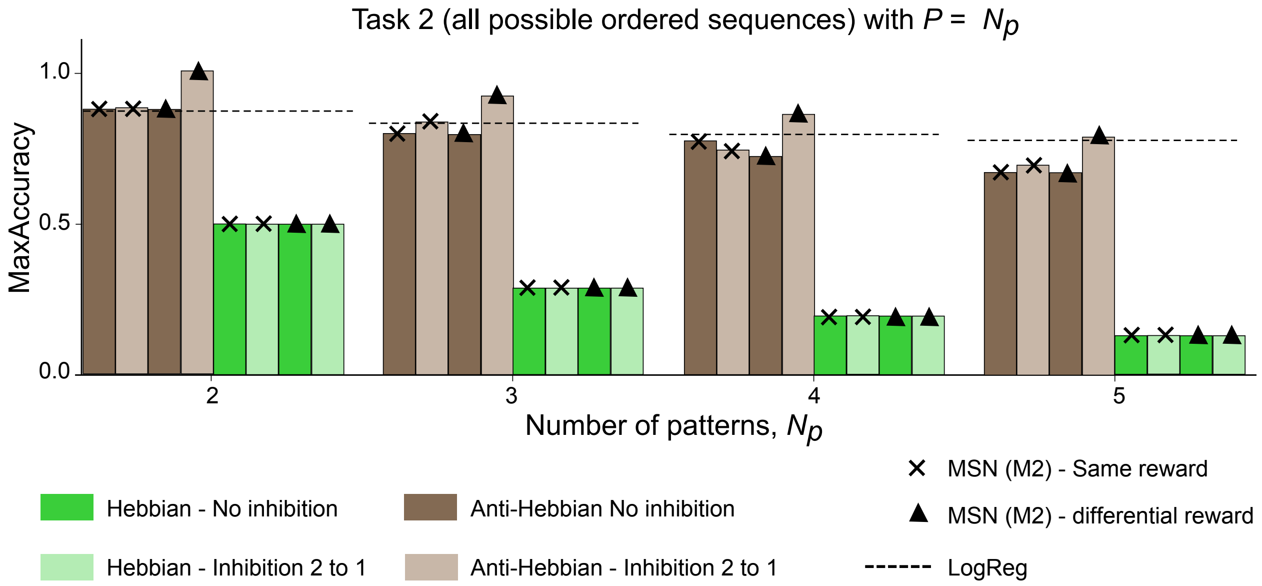

Figure S4: **Influence of the strategy of reward with collateral inhibition.** [Task 2] MaxAccuracy for different number of cortical neurons P, with N_p=P for different types of learning strategies and connectivity. (statistics and other parameters identical as in Figure 7)

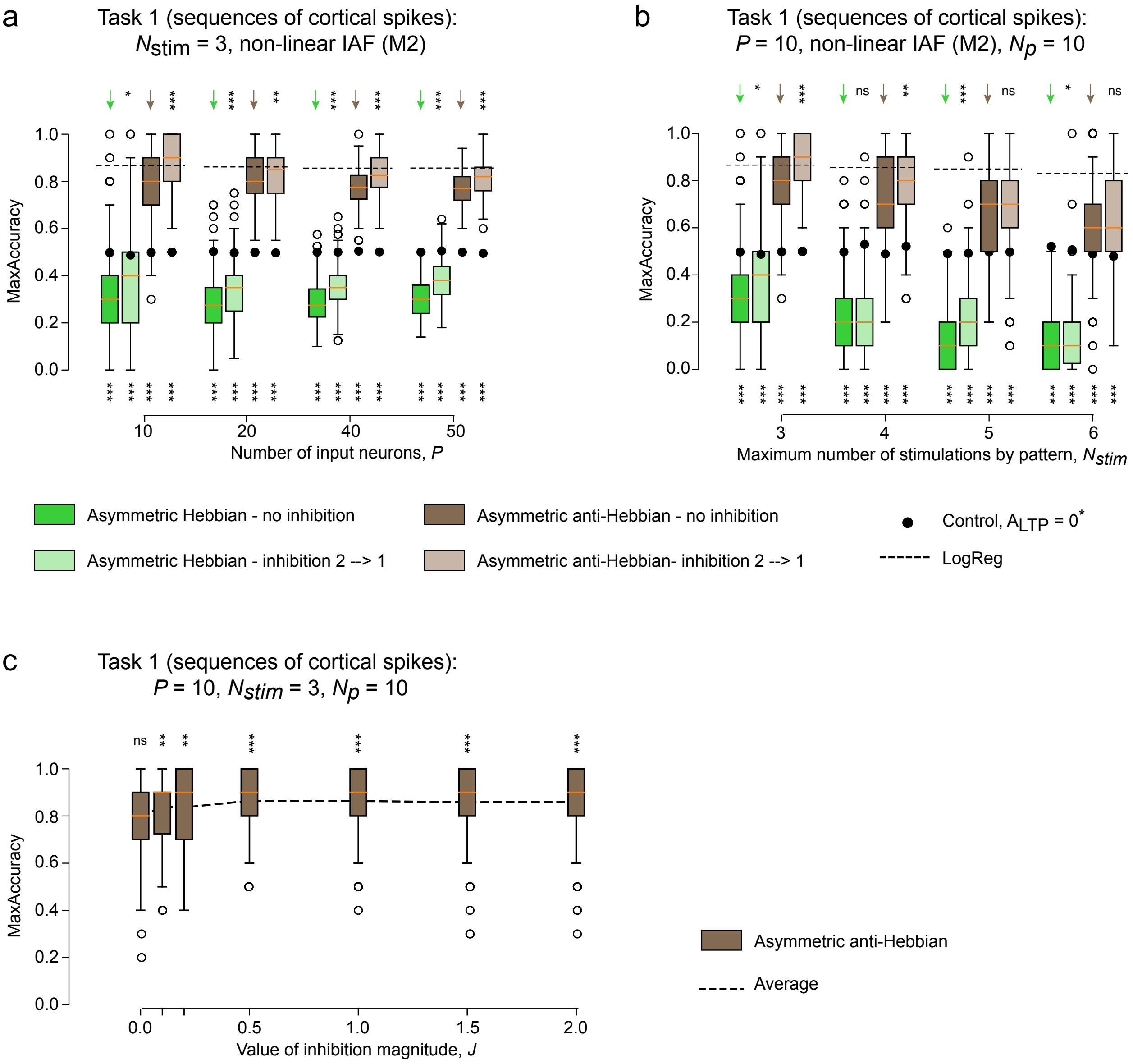

Figure S5: **Influence of parameters on learning with non-linear IAF model (M2) and lateral inhibition.** [Task 1] (a) MaxAccuracy for different number of cortical neurons P, with N_p=P. Training done for [500, 1000, 2000, 2000] patterns iterations for P=[10, 20, 40, 50], with test sessions every N_p=P iterations. (b) MaxAccuracy for different numbers of cortical stimulations N_{stim}. (c) MaxAccuracy for different values of lateral inhibition J for asymmetric anti-Hebbian STDP. (statistics and other parameters identical as in Figure 7).

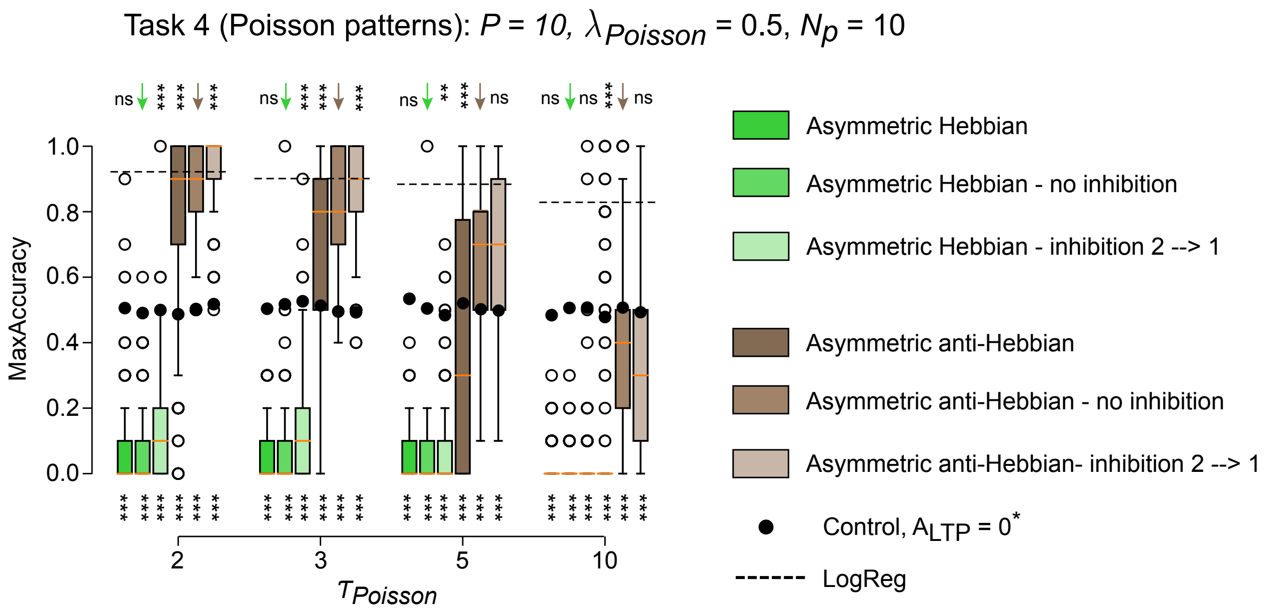

Figure S6: **Influence of pattern duration on sequence learning.** MaxAccuracy for model M1 (circles) and M2 (triangles), for a single neuron or a pair of neurons with inhibition, for the learning of $N_p = 10$ patterns from $P = 10$ input neurons, generated from a Poisson process with rate 0.5 kHz and various pattern durations (2, 3, 5 and 10 ms). Hebbian STDP shows no learning while anti-Hebbian STDP shows impaired learning with increased pattern duration.

Supplementary References

[1] Man Yi Yim, Ad Aertsen, and Arvind Kumar. Significance of Input Correlations in Striatal Function. *PLoS Computational Biology*, 7(11), November 2011. DOI: 10.1371/journal.pcbi.1002254.

[2] Eugene M. Izhikevich. *Dynamical Systems in Neuroscience*. MIT Press, 2007.
